# Susceptibility to innate immune activation in genetically mediated myocarditis

**DOI:** 10.1172/JCI180254

**Published:** 2024-05-16

**Authors:** Daniel F. Selgrade, Dominic E. Fullenkamp, Ivana A. Chychula, Binjie Li, Lisa Dellefave-Castillo, Adi D. Dubash, Joyce Ohiri, Tanner O. Monroe, Malorie Blancard, Garima Tomar, Cory Holgren, Paul W. Burridge, Alfred L. George, Alexis R. Demonbreun, Megan J. Puckelwartz, Sharon A. George, Igor R. Efimov, Kathleen J. Green, Elizabeth M. McNally

**Affiliations:** 1 Center for Genetic Medicine and; 2 Bluhm Cardiovascular Institute, Department of Medicine, Division of Cardiology, Northwestern University Feinberg School of Medicine, Chicago, Illinois, USA.; 3 Department of Biomedical Engineering, Northwestern University, Chicago, Illinois, USA.; 4 Department of Biology, Furman University, Greenville, South Carolina, USA.; 5 Department of Pathology,; 6 Department of Pharmacology,; 7 Department of Dermatology, and; 8 R.H. Lurie Comprehensive Cancer Center, Northwestern University Feinberg School of Medicine, Chicago, Illinois, USA.

**Keywords:** Cardiology, Inflammation, Cell stress, Cytokines, Genetic diseases

## Abstract

Myocarditis is clinically characterized by chest pain, arrhythmias, and heart failure, and treatment is often supportive. Mutations in *DSP*, a gene encoding the desmosomal protein desmoplakin, have been increasingly implicated in myocarditis. To model *DSP*-associated myocarditis and assess the role of innate immunity, we generated engineered heart tissues (EHTs) using human induced pluripotent stem cell–derived cardiomyocytes (hiPSC-CMs) from patients with heterozygous *DSP* truncating variants (*DSP*tvs) and a gene-edited homozygous deletion cell line (*DSP^–/–^*). At baseline, *DSP^–/–^* EHTs displayed a transcriptomic signature of innate immune activation, which was mirrored by cytokine release. Importantly, *DSP^–/–^* EHTs were hypersensitive to Toll-like receptor (TLR) stimulation, demonstrating more contractile dysfunction compared with isogenic controls. Relative to *DSP^–/–^* EHTs, heterozygous *DSP*tv EHTs had less functional impairment. *DSP*tv EHTs displayed heightened sensitivity to TLR stimulation, and when subjected to strain, *DSP*tv EHTs developed functional deficits, indicating reduced contractile reserve compared with healthy controls. Colchicine or NF-κB inhibitors improved strain-induced force deficits in *DSP*tv EHTs. Genomic correction of *DSP* p.R1951X using adenine base editing reduced inflammatory biomarker release from EHTs. Thus, EHTs replicate electrical and contractile phenotypes seen in human myocarditis, implicating cytokine release as a key part of the myogenic susceptibility to inflammation. The heightened innate immune activation and sensitivity are targets for clinical intervention.

## Abstract 2

A human engineered heart tissue model of *DSP*-associated myocarditis demonstrates baseline and triggered innate immune activation linked to contractile dysfunction and arrhythmias.

## Graphical abstract

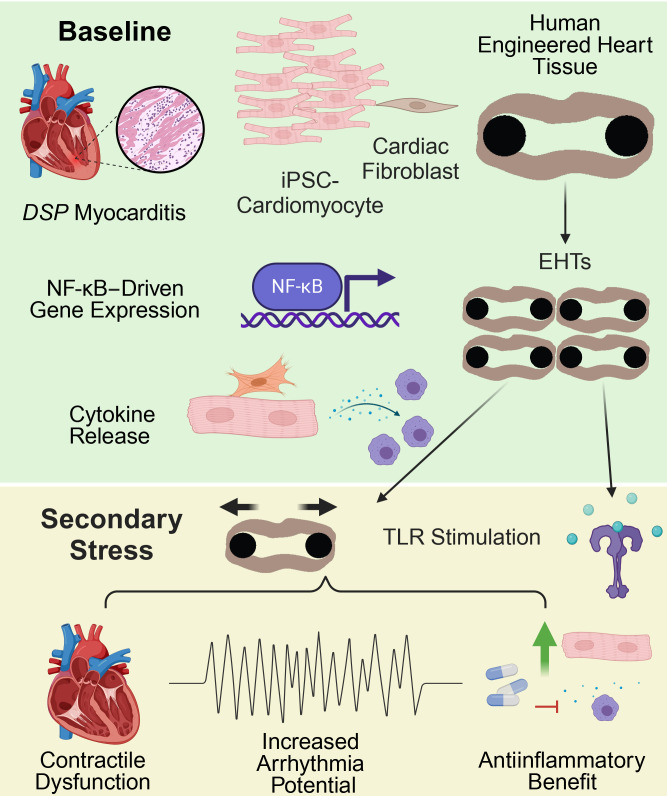

## Introduction

Myocarditis is a life-threatening heart condition characterized by inflammation, cellular infiltrate, chest pain, reduced heart function, and arrhythmias (1). Myocarditis affects as many as 10–20 per 100,000 people, with the highest incidence in men between 22 and 40; myocarditis can cause sudden cardiac death, and it is a common cause for cardiac transplant in young people. Clinically, myocarditis often comes on acutely, and it has both genetic and environmental triggers, including viral infections or response to vaccination (2). In recent years, pathogenic variants in *DSP*, the gene encoding desmoplakin, have been described in patients presenting with clinical myocarditis (2, 3). Although additional cardiomyopathy gene mutations have been described in these myocarditis case series, *DSP* is the most common gene reported, especially among those with relatively intact left ventricular function (4). These findings have prompted the idea that cardiomyopathy genetic testing is useful for persons with myocarditis and identifies those at risk for recurrence and additional sequelae of myocarditis, like heart failure and arrhythmias (4, 5).

The *DSP* gene encodes desmoplakin, a critical protein that forms an intracellular link connecting transmembrane cadherins and associated proteins to the cytoplasmic intermediate filament cytoskeleton as part of desmosomes (6). Desmosomal gene mutations are best known for their role in arrhythmogenic right ventricular cardiomyopathy (ARVC) (7). In ARVC, the desmosomal gene plakophilin 2 (*PKP2*) is the dominant ARVC-associated gene, with other desmosomal genes being much less represented (8). Studies evaluating the clinical findings in patients with *DSP* gene mutations reported that pathogenic *DSP* gene mutations are much more associated with left ventricular cardiomyopathy and a heightened arrhythmia risk, distinct from *PKP2*, which targets the right ventricle, causing classical ARVC (9). Heightened inflammation has been linked to the fibrofatty changes seen in ARVC (10), but the degree to which this process is similar in left ventricular *DSP*-associated cardiomyopathy and myocarditis is inadequately studied.

In *DSP* gene mutation carriers, cardiac inflammation is detected using cardiac imaging, specifically 18F-fluorodeoxyglucose positron emission tomography (9), and this inflammatory signature has been referred to as a “sterile” myocarditis-like process. Similar cardiac inflammation can be visualized in myocarditis patients with *DSP* gene mutations (11–22). In patients, the *DSP* mutations are present throughout their lifetimes, suggesting additional stressors contribute to manifesting myocarditis episodes, which histopathologically are characterized by myocardial necrosis and immune cell, typically lymphocytic, infiltrate. Defining the cardiomyocyte aspects of myocarditis susceptibility and understanding the potential triggers of myocarditis could help inform therapeutic strategies.

As a structural component of the desmosome, desmoplakin interacts with intermediate filaments, plakoglobin, and plakophilin, as well as other elements that directly and indirectly regulate electrical conduction in cardiomyocytes (23, 24). Reduction of *DSP* expression and *DSP* mutations are associated with reduced membrane targeting of connexin-43 (Cx43, *GJA1*) (25, 26). In the absence of *DSP*, there is an activation of ERK1/2–MAPK stress signaling, which leads to increased phosphorylation of Cx43, subsequently triggering degradation of gap junctions via lysosomes (6). In humans, heterozygous *PKP2* and *DSG2* mutations lead to ARVC, but in mice, homozygous deletion of *Dsg2* and *Pkp2* in the mouse heart is required to elicit cardiomyopathy and to define NF-κB activation, since heterozygous deletion has little phenotype in mice (27–30). Global deletion of *Dsp* in mice is lethal, so cardiomyocyte-specific homozygous *Dsp* has been used to study the cardiac effects of desmoplakin. Cardiomyocyte-specific *Dsp* deletion in mice produces cardiac dysfunction, arrhythmias, and fibrosis (31, 32), with activation of apoptosis and increased TGF-β and inflammatory pathways seen in gene expression signatures (33, 34).

Engineered heart tissues (EHTs) generated from human induced pluripotent stem cell–derived cardiomyocytes (hiPSC-CMs) can serve as useful models for human pathobiology in a 3D organoid format (35, 36). Compared with hiPSC-CMs grown in monolayers in the dish, an advantage of bioengineered platforms like EHTs is the alignment of cardiomyocytes, which promotes the formation of intercellular junctions (37, 38). EHT modeling of *DSP-*related cardiomyopathy previously demonstrated that dynamically shifting preload and afterload enhanced contractile defects (39). Ng et al. evaluated *DSP* R451G and 3 other missense variants and showed an increase in their sensitivity to calpain-mediated degradation (40), and this variant was evaluated in mice (41). To study both the alterations in contractility and arrhythmia propensity of *DSP* in a human organoid context, we generated patient-derived heterozygous *DSP* EHTs and used gene editing to create a homozygous *DSP^–/–^* line. We found that heterozygous patient-derived *DSP* EHTs had less contractile deficit at baseline but that these EHTs had impaired contractile reserve and enhanced susceptibility to innate immune activation. Stimulation of TLR pathways elicited contractile deficits like those seen in sterile myocarditis along with cytokine secretion, which in vivo would be expected to attract lymphocytes and macrophages, creating a feed forward cycle of myocardial injury and arrhythmias. Blocking NF-κB using BAY 11-7082 or colchicine effectively suppressed cytokine release and restored *DSP*-induced contractile deficits. When applied to *DSP*-associated myocarditis, these findings support an acute-on-chronic model of innate immune activation that is targetable using blockers of innate immunity.

## Results

### DSP truncations and clinical presentations.

**Figure 1:**
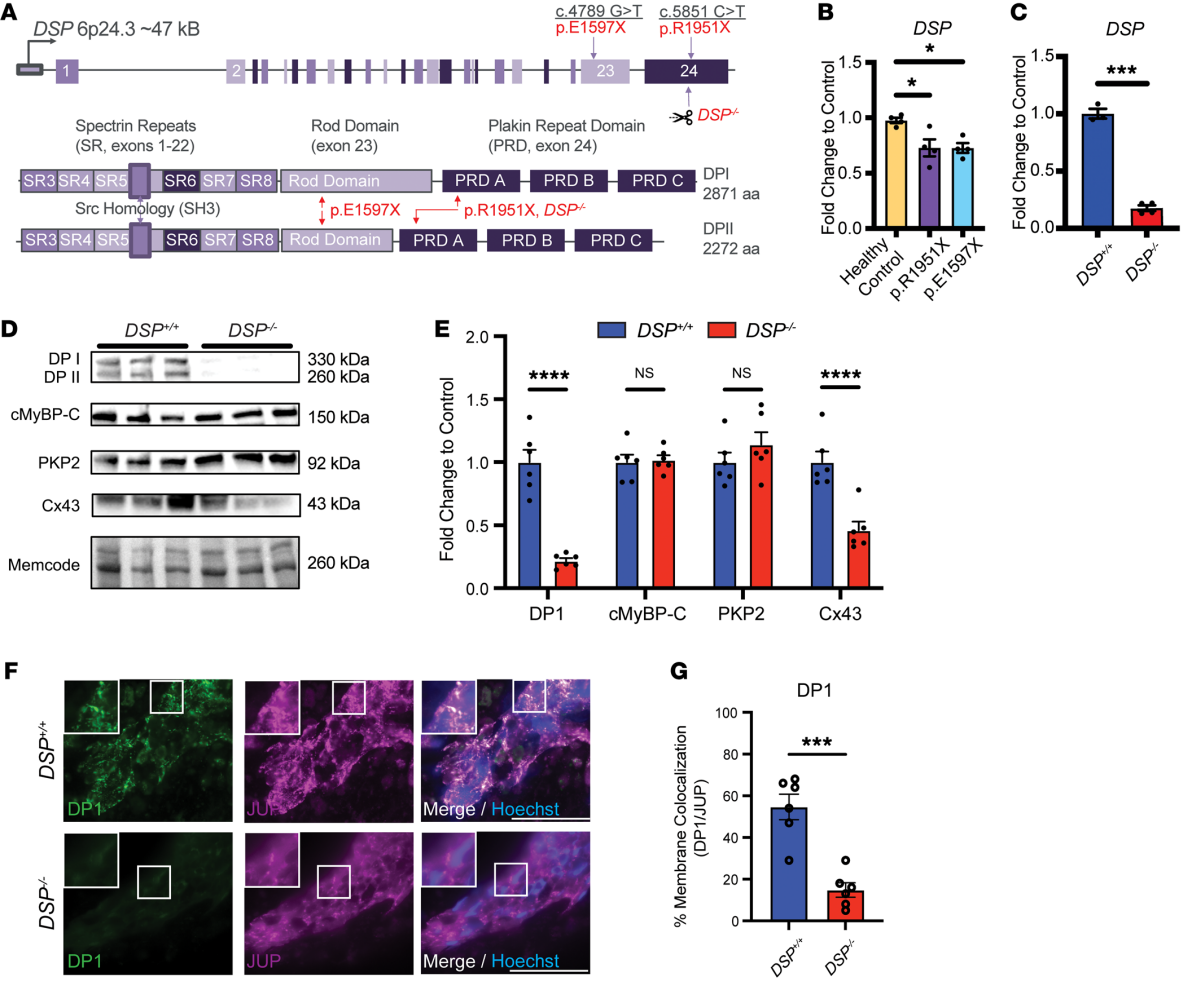
EHTs with DSP truncating variants. (**A**) The *DSP* locus includes 24 exons and is alternatively spliced to produce DPI and DPII. Heterozygous *DSP* nonsense mutations p.E1597X and p.R1951X fall within the rod domain (exon 23) and plakin repeat domain (exon 24), respectively. *DSP* p.E1597X was identified in a young woman presenting with classical myocarditis while the individual with *DSP* p.R1951X had a family history of myocarditis. Scissors mark the site of CRISPR/Cas9 guides used to generate *DSP^–/–^* homozygous hiPSCs. Homozygous *DSP*^–/–^ EHTs were generated to compare severity with the patient-derived heterozygous *DSP* truncations. (**B**) *DSP* transcript was reduced in *DSP* p.R1951X and p.E1597X heterozygous patient cardiomyocytes compared with healthy control (*<0.05 by 2-way ANOVA; *n* = 4 per condition; data reflect 2 independent differentiations). (**C**) *DSP* mRNA was reduced in *DSP^–/–^* EHTs compared with isogenic control EHTs (***< 0.001 by 2-tailed *t* test with Welch’s correction; *n* = 3 *DSP^+/+^* and *n* = 4 *DSP^–/–^*; data reflect 2 independent differentiations). (**D** and **E**) Immunoblot of *DSP^–/–^* hiPSC-CMs demonstrated substantial loss of DPI, DPII, and connexin 43 (Cx43) staining compared with isogenic controls (*DSP^+/+^*). Staining of desmosome component PKP2 and sarcomere component cMyBP-C demonstrated no significant difference between lines as quantified in **E** (****< 0.0001 by 2-way ANOVA; *n* = 6 *DSP^+/+^* and *n* = 6 *DSP^–/–^*; data reflect 3 independent differentiations). (**F**) Representative immunofluorescence imaging of *DSP^+/+^* and *DSP^–/–^* EHTs staining for DPI (green) and desmosome component plakoglobin (JUP, purple). Nuclei appear blue. Scale bars represent 100 μm. All insets are 100× original magnification. Insets depict intercalated disc–like structures with lower membrane localization of DPI in *DSP^–/–^* EHTs compared with control as measured by colocalization with JUP (**G**) (***< 0.001 by 2-tailed *t* test with Welch’s correction; *n* = 6 per condition). All data presented as mean ± SEM.

To evaluate the role of *DSP* mutations in genetically mediated cardiomyopathy and myocarditis, we generated 2 patient-derived hiPSC lines for study, *DSP* c.4789G > T (p.E1597X) and *DSP* c.5851C > T (p.R1951X) (Supplemental Table 1; supplemental material available online with this article; https://doi.org/10.1172/JCI180254DS1; and Figure 1A). *DSP* p.E1597X was identified in a woman in her early 20s who was diagnosed with myocarditis after presenting with bradycardia and heart failure. A cardiac MRI showed diffuse delayed enhancement, and she was initially treated with pressor support and guideline-directed therapy for heart failure. Because of ongoing symptoms and nonsustained ventricular tachycardia (NSVT), she was also treated with colchicine, with improvement over several months. Genetic testing with a large gene panel showed a pathogenic heterozygous *DSP* truncation, p.E1597X. An hiPSC line was also generated from a woman in her 50s who was diagnosed with cardiomyopathy (left ventricular ejection fraction 40%–45%) and NSVT. She was identified as having a pathogenic heterozygous *DSP* truncation, p.R1951X, which was identified by cascade testing because her first-degree relative presented with myocarditis and ventricular tachycardia, as previously described (14). HiPSCs were differentiated to cardiomyocytes for 10 days and then placed into collagen hydrogels to generate EHTs composed of cardiomyocytes derived from hiPSC cell lines and human cardiac fibroblasts, and EHTs were maintained for 21–35 days before harvest and study (schematic and metrics shown in Supplemental Figures 1–3). Quantitative PCR of RNA using patient-derived EHTs with either p.E1597X or p.R1951X documented reduced expression of *DSP* mRNA compared with healthy control hiPSC-CMs, consistent with the heterozygous loss of function in *DSP* pathological genetic variants (Figure 1B) via nonsense-mediated decay as suggested by a meta-analysis of *DSP* cardiomyopathic mutations and outcomes (42).

To generate a more severe model of *DSP* myocarditis and cardiomyopathy with an isogenic control, we used a healthy control hiPSC line to generate biallelic *DSP* truncations with CRISPR/Cas9, referred to as *DSP*^–/–^. The guide RNAs used in gene editing targeted exon 24 in a region near p.R1951X (Figure 1A and Supplemental Figure 4). We identified a compound heterozygous *DSP* line (Supplemental Table 2), and this line had no documented off-target editing (Supplemental Table 3); this line was termed *DSP^–/–^*, and its isogenic healthy control line is referred to as *DSP^+/+^*. *DSP* mRNA was significantly reduced in EHTs generated from the *DSP^–/–^* line (Figure 1C). *DSP^–/–^* EHTs had correspondingly reduced *DSP* protein (isoforms DPI and DPII) with relatively normal myosin binding protein C (cMyBP-C) and PKP2 protein levels, while Cx43 protein was reduced in *DSP^–/–^* EHTs when compared with the isogenic control line, *DSP^+/+^* (Figure 1, D and E). In the EHT format, *DSP^+/+^* EHTs formed immature intercalated disc–like structures, which were not as well formed in *DSP^–/–^* EHTs. Junctional plakoglobin (JUP) appeared similar between *DSP^+/+^* and *DSP^–/–^* EHTs; colocalization studies of JUP with DSP protein highlighted the differences in the intercalated disc–like structures between *DSP^–/–^* and *DSP^+/+^* EHTs (Figure 1, F and G). The reduction in Cx43 and less well-formed intercalated disc–like structures suggested that the *DSP^–/–^* EHTs have features consistent with arrhythmia-prone substrates (25, 31).

### Contractile and electrical defects in DSP^–/–^ EHTs.

**Figure 2:**
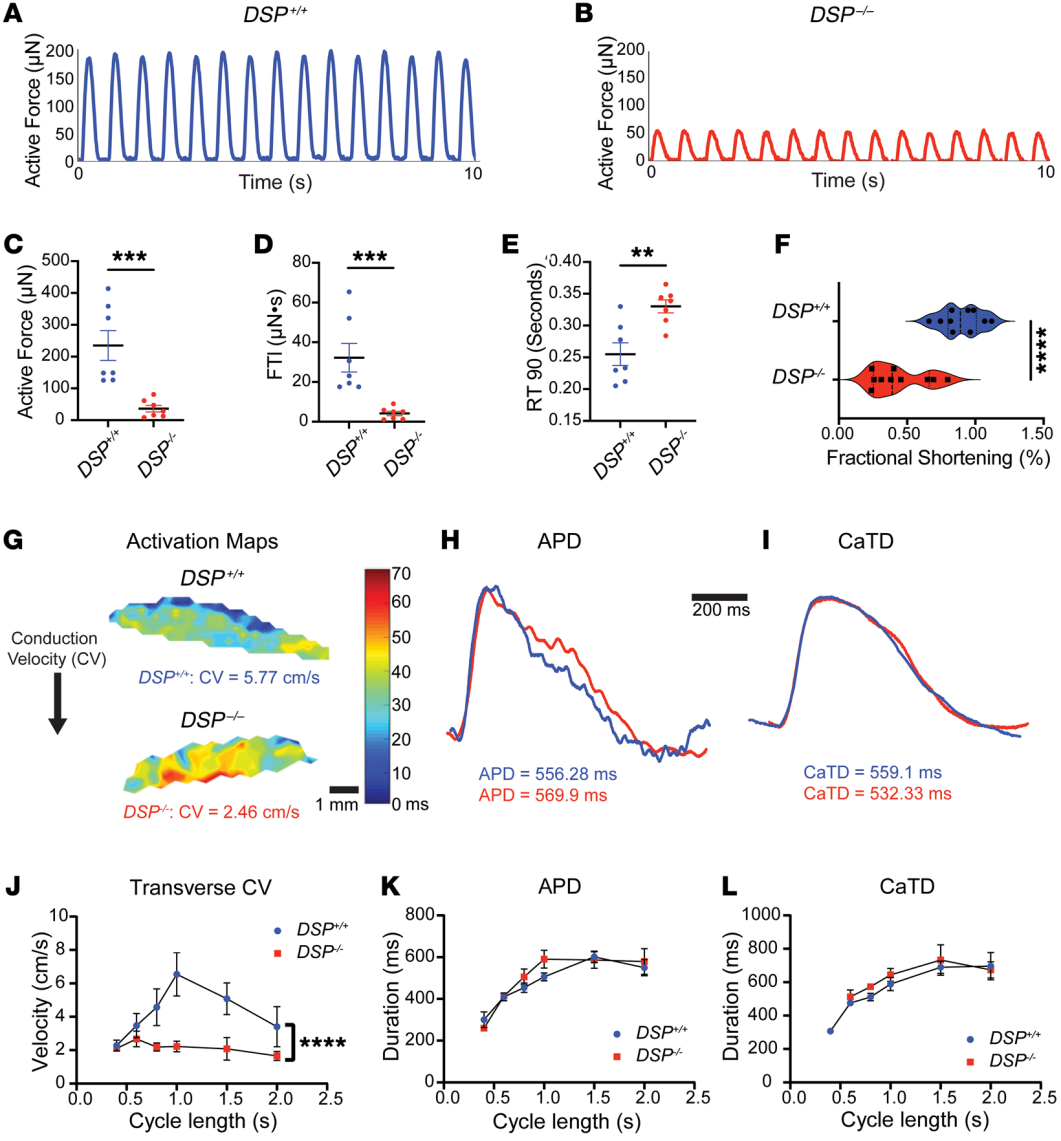
Mechanical and electrical defects in *DSP*^–/–^ EHTs. (**A** and **B**) Active force of *DSP*^–/–^ and isogenic *DSP*^+/+^ control EHTs recorded at baseline. (**C** and **D**) Peak force and force time integral (FTI) were significantly reduced in *DSP*^–/–^ EHTs compared with *DSP*^+/+^ EHTs. (**E**) The time to 90% force reduction from peak (RT 90) was prolonged in *DSP*^–/–^ compared with *DSP*^+/+^ EHTs. (***< 0.001, **< 0.01 by Mann-Whitney *U* test; *n* = 7 *DSP*^+/+^ and *n* = 7 DSP^–/–^; data reflect 7 independent batches.) (**F**) Fractional shortening (FS) of *DSP*^–/–^ EHTs was significantly reduced at baseline (****< 0.0001 by 2-tailed *t* test with Welch’s correction; *n* = 10 *DSP*^+/+^ and *n* = 10 *DSP^–/–^*; data reflect 3 independent batches). (**G**) Optical mapping of *DSP*^+/+^ and *DSP*^–/–^ EHTs at cycle length of 1 second measuring transverse conduction velocity (CV) with representative action potential duration (APD) (**H**) and Ca^2+^ transient duration (CaTD) (**I**). (**J**–**L**) Restitution curves of *DSP*^–/–^ and *DSP*^+/+^ EHTs demonstrated significant reduction of CV (**J**) with no difference in APD (**K**) and CaTD (**L**) across cycle lengths (****< 0.0001 by nonlinear regression analysis, *n* = 4–6 EHTs per condition across 3 batches). All data presented as mean ± SEM.

To assess the mechanical consequences in *DSP^–/–^* EHTs, we measured force production compared with isogenic *DSP^+/+^* EHTs under pacing conditions. Active force was reduced in *DSP^–/–^* EHTs from mean ± SEM 237 ± 118 μN for *DSP^+/+^* EHTs to 36 ± 26 μN (*P* = 0.0006) (Figure 2C). Furthermore, the force time integral (FTI) was reduced from 35 ± 18 μN•s in *DSP^+/+^* EHTs to 4.0 ± 3.0 μN•s (*P* = 0.0006), and the time to 90% reduction in peak force was slowed in *DSP^–/–^* EHTs to 0.33 ± 0.04 seconds versus 0.25 ± 0.05 seconds in *DSP^+/+^* EHTs (*P* = 0.007) (Figure 2, D and E). Fractional shortening of spontaneously beating EHTs was significantly reduced in beating *DSP^–/–^* EHTs compared with beating *DSP^+/+^* EHTs (*P* < 0.0001) (Supplemental Table 4 and Figure 2F). To characterize the electrical properties of *DSP^–/–^* EHTs, we conducted optical mapping studies to evaluate conduction velocity (CV), action potential duration (APD), and Ca^2+^ transient duration (CaTD) (Figure 2). Optical mapping and restitution curve analysis demonstrated that *DSP^–/–^* EHTs had slower CV (Figure 2, G and J), seen across a range of cycle lengths, while APD (Figure 2, H and K) and CaTD (Figure 2, I and L) were not appreciably different between *DSP^–/–^* and *DSP^+/+^* EHTs. These findings likely reflect reduced Cx43, and taken together, these data indicated a role for *DSP* in both mechanical and electrical function, specifically in slowing CV.

### Activated inflammation in DSP^–/–^ EHTs.

**Figure 3:**
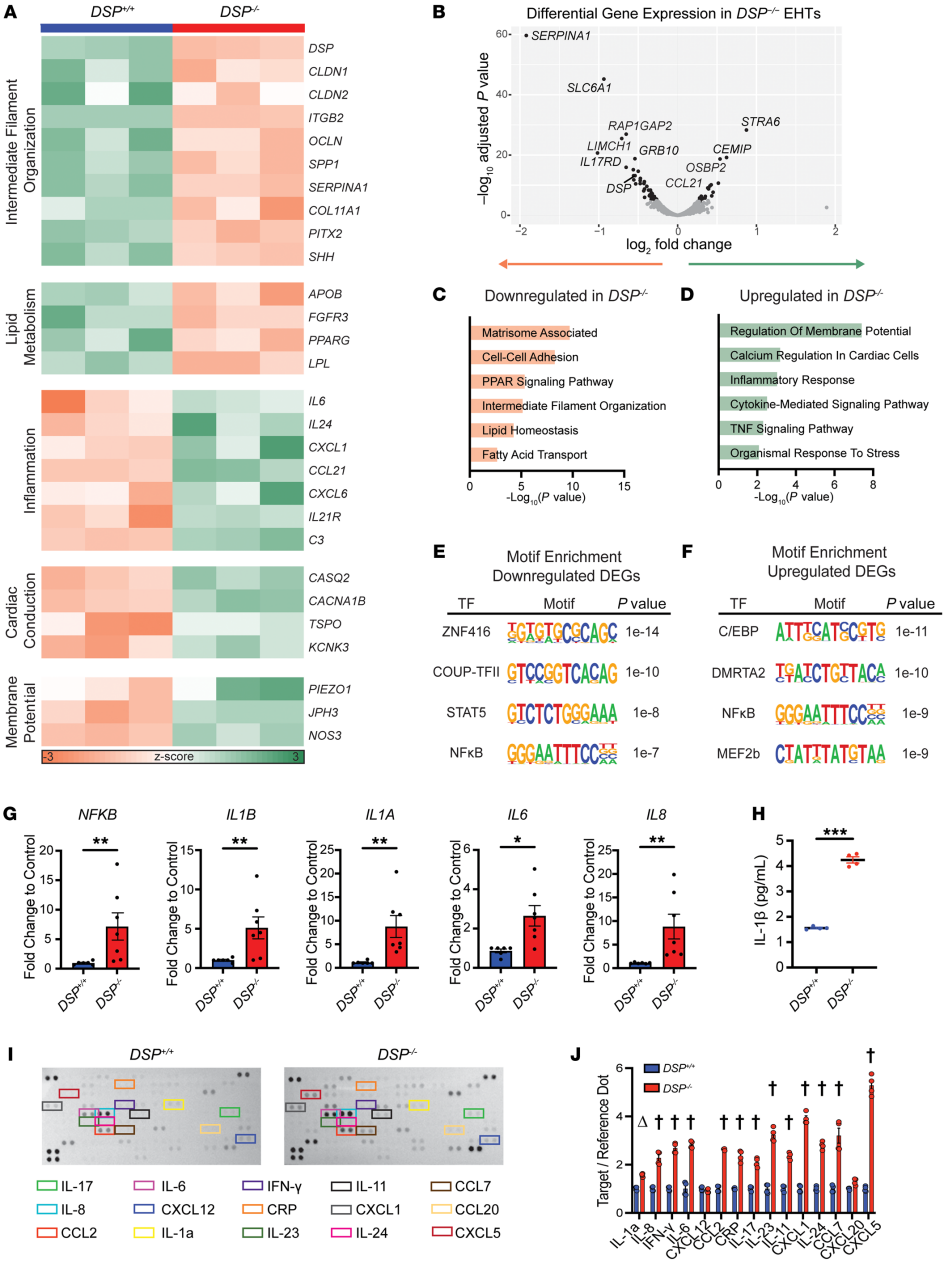
Inflammatory signatures in gene expression and cytokine release from *DSP^–/–^* EHTs. (**A**–**D**) RNA-Seq analysis of *DSP^+/+^* and *DSP^–/–^* EHTs identified differentially expressed genes (DEGs). (**A**) Heatmap of selected transcripts associated with Gene Ontology (GO) terms in *DSP^–/–^* compared with *DSP*^+/+^ EHTs. (**B**) Volcano plot of RNA-Seq showing significant DEGs between *DSP^–/–^* and *DSP*^+/+^ EHTs, highlighting the top transcripts by *P* value (gray *P* > 0.05, black *P* < 0.05). (**C** and **D**) GO term analysis shows downregulation of intermediate filament organization and lipid metabolism terms (**C**) and upregulation of inflammation, cardiac conduction, and membrane potential terms (**D**) in *DSP^–/–^* EHTs compared with control. (**E** and **F**) Motif enrichment analysis of the top 300 DEGs identified transcription factor (TF) motifs associated with downregulated DEGs (**E**) and upregulated DEGs (**F**) in *DSP^–/–^* compared with *DSP*^+/+^ EHTs. Motifs depicted represent consensus sequences to which DEGs aligned with *P* value for enrichment. (**G**) RT-qPCR demonstrated an increase in inflammatory transcripts in *DSP^–/–^* compared with *DSP*^+/+^ (**< 0.01, *< 0.05 by Mann-Whitney *U* test; *n* = 6 *DSP*^+/+^ and *n* = 7 *DSP^–/–^*, 3 independent batches). (**H**) ELISA of IL-1β in EHT media demonstrated increased IL-1β in *DSP^–/–^* compared with *DSP*^+/+^ EHT media (***< 0.0001 by 2-tailed *t* test with Welch’s correction; *n* = 4 *DSP*^+/+^ and *n* = 4 DSP^–/–^). (**I**) Representative cytokine arrays were incubated with EHT media. The corner signals serve as reference markers (Reference Dots). Targets displayed in colored boxes are quantified (**J**) (^Δ^< 0.01, ^†^< 0.0001 by 2-way ANOVA with *n* = 4 *DSP*^+/+^ and *n* = 4 *DSP^–/–^*; data reflect 2 independent batches). All data presented as mean ± SEM.

To identify molecular pathways disrupted in *DSP^–/–^* EHTs, we performed RNA sequencing (RNA-Seq) on intact EHTs and found transcriptomic changes contributing to mechanical and electrical dysfunction (Figure 3). GO pathway analysis verified intermediate filament downregulation driven by loss of *DSP* (Figure 3A). The 281 downregulated genes and 160 upregulated genes in *DSP^–/–^* EHTs compared with *DSP^+/+^* EHTs (FDR ≤ 0.05, log counts per million ≥ 6.25, Figure 3B) mapped to dysregulated GO pathways including downregulation of lipid metabolism and upregulation of inflammation, cardiac conduction, and membrane potential genes (Figure 3, C and D, with select individual genes shown in Figure 3A). Downregulation of genes for matrisome-associated and cell-cell adhesion pathways was a feature of *DSP^–/–^* EHTs consistent with disruption of intercellular and cell-matrix attachments as consequences of desmosomal disruption.

We evaluated TF motifs enriched near upstream transcriptional start sites of DEGs in *DSP^–/–^* compared with *DSP^+/+^* EHTs (Figure 3, E and F). Among upregulated DEGs, motifs for the TFs C/EBP, DMRTA2, and MEF2b were enriched. For downregulated DEGs, enriched TF motifs included ZNF416, COUP-TFII, and STAT5 recognition sites. ZNF416 is a zinc finger nuclease associated with fibroblast activation (43), and COUPTFII, encoded by the gene *NR2F2*, has been reported to repress glucocorticoid transcriptional activity and alter cardiac metabolism in heart failure (44). The downregulation of STAT5 and inclusion of NF-κB motifs in both downregulated and upregulated genes are consistent with profound dysregulation of innate immunity. MEF2b sites were associated with increased gene expression in *DSP^–/–^* EHTs, as were DMRTA2 sites, an important factor in the neuronal lineage, consistent with pathologic perturbation of cell type specification.

Since EHTs are composed of both hiPSC-CMs and cardiac fibroblasts at a 9:1 ratio, we evaluated whether siRNA knockdown of *Dsp* in cardiomyocytes alone was sufficient to replicate transcriptional changes observed in EHTs. siRNA knockdown of *Dsp*, *Pkp2*, and *Jup* in neonatal rat ventricular cardiomyocytes (NRVMs) revealed reduction of *Dsp* was associated with upregulation of innate immune pathways in NRVMs (Supplemental Figure 5). Interestingly, NRVMs treated with siRNAs to *Pkp2* or *Jup* did not display the same upregulation of innate immune pathway genes. This differential upregulation might derive from degree of primary RNA target reduction, intrinsic differences in biological processes, and/or differences between the right ventricle (RV) and left ventricle (LV), as NRVMs represent more LV than RV based on total heart mass. In hiPSC-CM–derived EHTs, we verified specific upregulation of genes associated with innate immune activation using RT-PCR, and these results demonstrated upregulation of *NFKB*, *IL1B*, *IL1A*, *IL6*, and *IL8* in *DSP^–/–^* EHTs compared with isogenic controls (Figure 3G). As a whole, gene expression changes in *DSP^–/–^* EHTs were consistent with a pathological role for activation of innate immunity and inflammation as a disease mechanism, mirroring myocarditis-like findings (9).

We interrogated whether the inflammatory gene signature in *DSP* EHTs was reflective of cytokine activation. We collected media from cultured *DSP^–/–^* and *DSP^+/+^* EHTs to evaluate cytokine release from EHTs into culture media. The immune activator IL-1β was increased in *DSP^–/–^* compared with *DSP^+/+^* EHTs (Figure 3H). We compared the cytokine profiles from *DSP^–/–^* and *DSP^+/+^* EHTs, finding a range of chemokines and cytokines elevated in *DSP^–/–^* culture media. We observed upregulation of Th17-dominant lymphocytic chemokines with increases in IL-17 and IL-23 (Figure 3, I and J). The downregulation of the *IL17RD* gene shown in Figure 3B was consistent with a compensatory response to higher levels of the circulating cytokine and increased activation of NF-κB (45). The upregulation of IL-6, IL-8, and IFN-γ suggested strong innate immune activation with the potential for adaptive immune cell recruitment with disruption of *DSP*.

### Innate immune activation exacerbates contractile dysfunction in DSP^–/–^ EHTs.

We evaluated whether immune activation in *DSP^–/–^* EHTs could be further stimulated by exposing EHTs to known activators of the TLR system to model myocarditis, albeit without lymphocytes and macrophages and as a means to examine the cardiomyocyte features that may underlie this process. Treatment with lipopolysaccharide (LPS), a component of Gram-negative bacterial cell membranes, was chosen to represent bacterial triggers of the innate immune system. High mobility group box protein 1 (HMGB1), a chromatin-associated protein released upon nuclear rupture, was chosen to represent stimulation of the TLR system via damage-associated molecular patterns that activate inflammation associated with wound healing. Of note, we verified that expression of pattern recognition receptors targeted by these TLR agonists did not demonstrate any significant difference in gene expression between *DSP^+/+^* and *DSP^–/–^* EHTs, suggesting that these signaling components remain functional in the context of *DSP* deficiency under the observed inflammatory response (Supplemental Figure 6, A–D). When treated with innate immune stimulants, inflammatory biomarkers were further elevated in *DSP^–/–^* EHTs compared with isogenic controls (Supplemental Figure 7, A–I), consistent with heightened sensitivity to an inflammatory environment.

**Figure 4:**
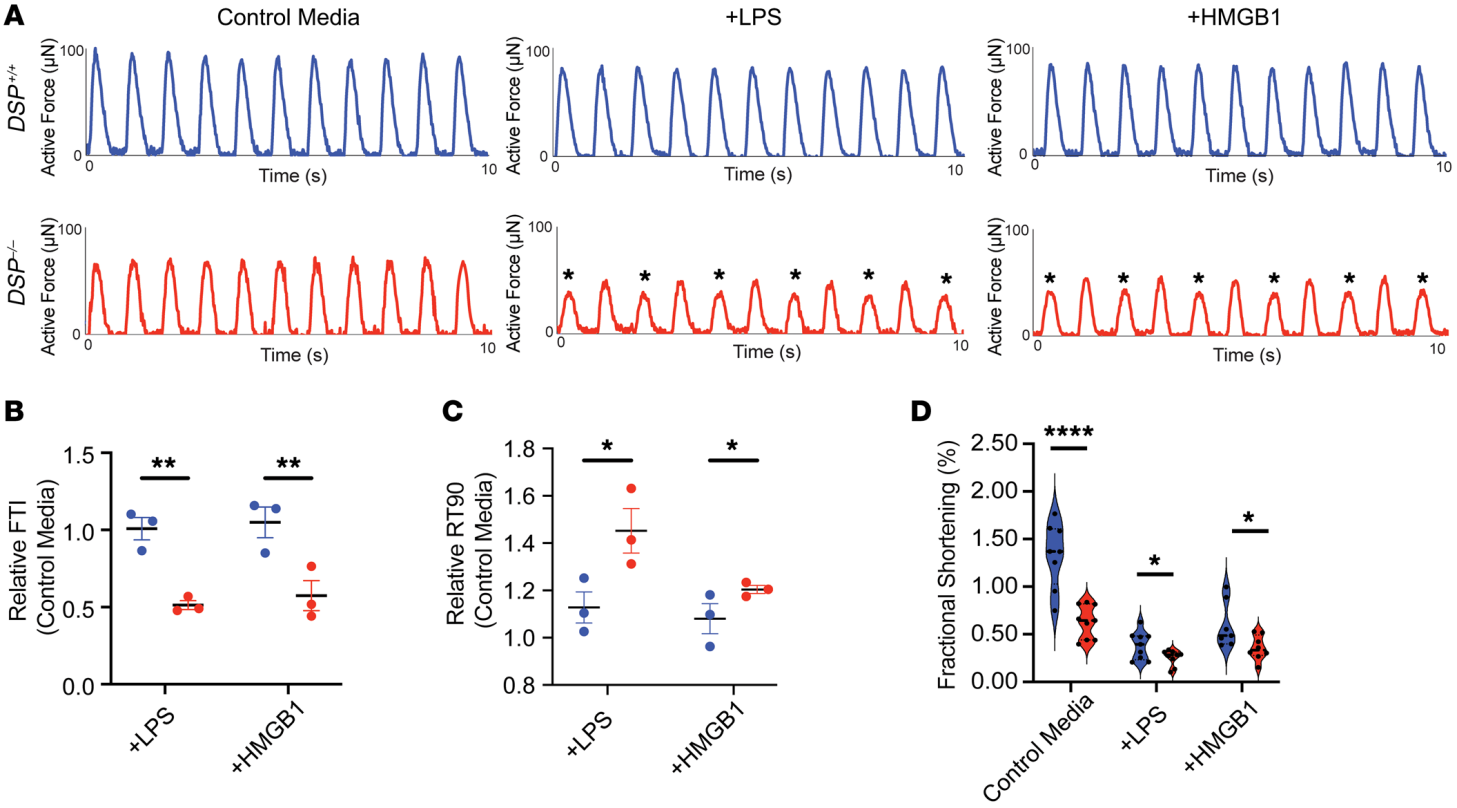
Innate immune activation exacerbates contractile deficits in *DSP^–/–^* EHTs. (**A**) Representative forces from *DSP*^+/+^ and *DSP^–/–^* EHTs recorded following 48 hours of exposure to normal media or media containing LPS or HMGB1. In *DSP^–/–^* EHTs, LPS and HMGB1 exposure markedly impaired force production and elicited alternans (marked with asterisks). (**B**) Relative to control media, *DSP^–/–^* EHTs had a greater reduction in FTI after LPS and HMGB1 exposure compared with *DSP*^+/+^ EHTs after similar exposure. (**C**) RT90 was also more prolonged in *DSP^–/–^* EHTs after LPS and HMGB1 compared with *DSP*^+/+^ EHTs cultured under inflammatory conditions (*< 0.05, **< 0.01 by 2-way ANOVA, *n* = 3 *DSP*^+/+^ and *n* = 3 *DSP^–/–^*; data reflect 3 independent batches). (**D**) Exposure to LPS or HMGB1 reduced fractional shortening in *DSP*^+/+^ and *DSP^–/–^* EHTs (*< 0.01, ****< 0.0001 by 2-tailed *t* test with Welch’s correction; *n* = 8 *DSP*^+/+^ and *n* = 8 *DSP^–/–^*; data reflect 3 independent differentiations). Data presented as mean ± SEM. Data presented as individual recordings normalized to average baseline measurement per EHT. Significance calculated based on mean value per EHT.

In response to LPS or HMGB1 stimulation, *DSP*^–/–^ EHTs, but not *DSP*^+/+^ EHTs, demonstrated marked alternans in which irregular peak force measurements were seen (Figure 4A, marked with asterisks). Measured as varying peak force values, we infer this variation to reflect arrhythmia propensity as it does in human premature ventricular beats (27). Multielectrode array studies of *DSP^–/–^* hiPSC-CM monolayers cultured on plates demonstrated an NF-κB–dependent increase in field potential duration (FPD) in response to HMGB1 (Supplemental Figure 8A). Direct force measurements showed greater reduction in FTI in *DSP^–/–^* EHTs compared with *DSP^+/+^* EHTs after LPS or HMGB1 (Figure 4B). When compared with EHTs in the absence of immune activation, *DSP^–/–^* EHTs treated with LPS and HMGB1 had an even greater prolongation of relaxation time than *DSP^+/+^* EHTs (Figure 4C), consistent with diastolic dysfunction. LPS and HMGB1 stimulation also decreased fractional shortening in EHTs compared with control media, measured as post deformation during contraction and relaxation (Figure 4D and Supplemental Table 5; LPS, *P* = 0.04; HMGB1, *P* = 0.03). These findings demonstrated enhanced susceptibility of *DSP^–/–^* EHTs to innate immune activation.

### Contractile properties of patient-derived heterozygous DSPtv EHTs.

**Figure 5:**
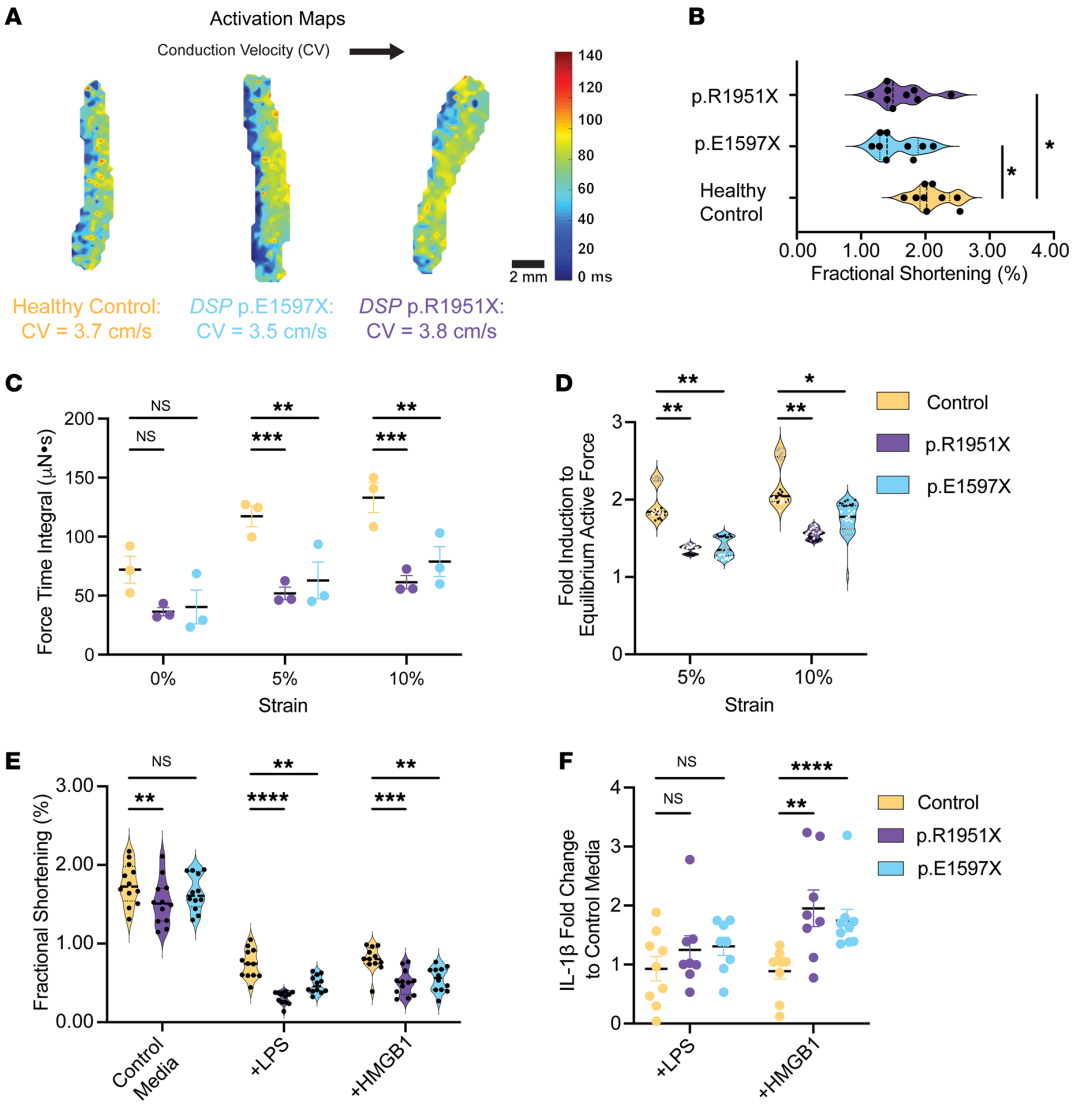
Strain-induced force loss in patient-derived *DSP* EHT models. (**A**) Optical mapping of heterozygous *DSP* p.E1597X and *DSP* p.R1951X EHTs compared with EHTs from healthy control demonstrates comparable conduction velocities, distinct from those seen in *DSP*^–/–^ EHTs. (**B**) Fractional shortening was only minimally reduced at baseline in *DSP* p.E1597X and p.R1951X EHTs compared with healthy control (*< 0.05 by 2-way ANOVA, *n* = 9–12 EHTs per condition, data representative of 3 batches). (**C** and **D**) FTI values from *DSP* p.E1597X and p.R1951X EHTs showed no significant difference in FTI compared with healthy control EHTs at baseline conditions. However, when subjected to 5% or 10% strain, both p.E1597X and p.R1951X EHTs failed to augment contraction as seen with healthy control EHTs (**D**), consistent with the requirement for additional stressors to manifest reduced contractility and reflecting the reduced contractile reserved of *DSP* heterozygous EHTs (*< 0.05, **< 0.01, ***< 0.001 by 2-way ANOVA, *n* = 3 EHTs per condition from 3 independent batches, labeled as n1 = black dots, n2 = white dots, n3 = gray dots). (**E**) After LPS or HMGB1 exposure, *DSP*tv EHTs had more marked reduced fractional shortening compared with similarly exposed healthy control EHTs (**< 0.01, ***< 0.001, ****< 0.0001 by 2-way ANOVA, *n* = 12 EHTs per condition). (**F**) LPS stimulated greater IL-1β release into the media compared with healthy control (**< 0.01, ****< 0.0001 by Mann-Whitney *U* test, *n* = 8 EHTs per condition). Data presented as individual recordings normalized to average baseline measurement per EHT. Significance calculated based on mean value per EHT.

We anticipated less severe but clinically relevant phenotypes in EHTs derived from patients with heterozygous *DSP* truncation variants (*DSP*tvs). The slowing of CV seen in *DSP^–/–^* EHTs was not evident in *DSP*tv EHTs (Figure 5A). Compared with *DSP^–/–^* EHTs, *DSP* p.R1951X and p.E1597X had a milder reduction of fractional shortening (Figure 5B and Supplemental Table 4). We evaluated length-dependent activation under isometric conditions as a proxy for contractile reserve since contractile reserve is typically reduced in patients with cardiomyopathy. At culture length (0%), force production in patient-derived *DSP*tv EHTs was comparable to healthy control EHTs, measured as FTI (Figure 5C). However, when lengthened to create more strain mimicking increased preload, *DSP* p.R1951X and p.E1597X EHTs failed to augment force production like healthy control EHTs, reflecting an impairment in contractile reserve (Figure 5D).

Innate immune activation with exposure to either LPS or HMGB1 reduced fractional shortening more in patient-derived *DSP*tv EHTs than healthy control EHTs for both p.R1951X and p.E1597X EHTs (Figure 5E and Supplemental Table 5), consistent with enhanced susceptibility to innate immune activation. The inflammatory biomarker IL-1β was increased in the media when *DSP*tv EHTs were treated with HMGB1, while healthy control EHTs did not have a change in IL-1β level in response to LPS (Figure 5F). Together, these findings showed a susceptibility of *DSP*tv EHTs to innate immune activation with cytokine release and a negative effect on contractility.

### NF-κB signaling drives contractile dysfunction in DSPtv EHTs.

**Figure 6:**
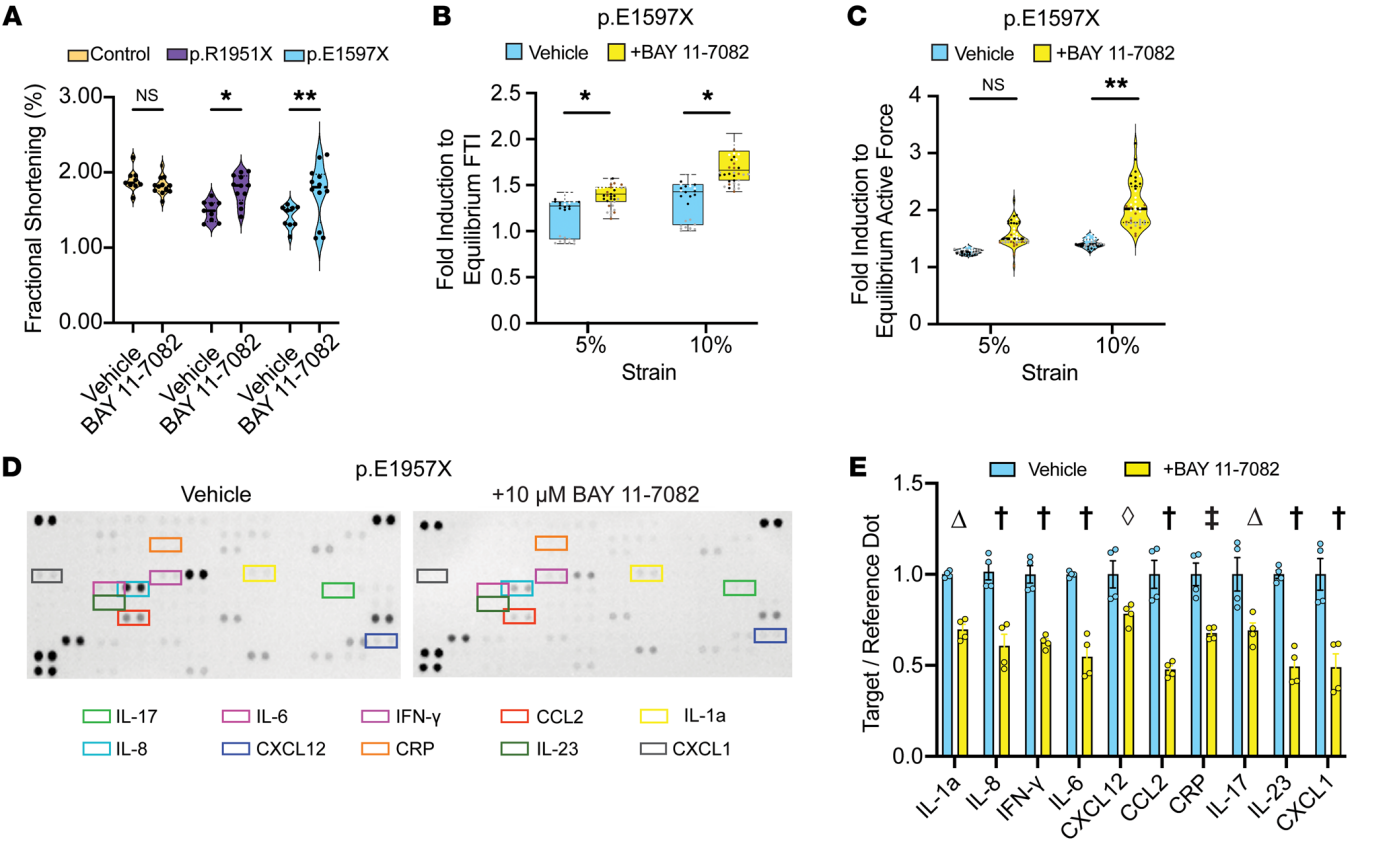
Improved contractile function with NF-κB inhibition in *DSP* EHTs. (**A**) Treatment with BAY 11-7082, an NF-κB inhibitor, improved fractional shortening of *DSP* p.E1597X and p.R1951X EHTs with no significant effect on healthy control cells (*< 0.05, **< 0.01 by 2-way ANOVA, *n* = 9–12 EHTs per condition, data representative of 3 batches). (**B** and **C**) BAY 11-7082 improved strain-induced force loss in *DSP* p.E1597X EHTs as measured in force time integral (**B**) and active force (**C**) (*< 0.05, **< 0.01 by 2-way ANOVA, *n* = 3–4 EHTs per condition across 2 batches, labeled as n1 = black dots, n2 = white dots, n3 = gray dots, n4 = brown dots). Box plots show the interquartile range, median (line), and minimum and maximum (whiskers). (**D** and **E**) Cytokine arrays of p.E1597X EHT media showed a significant reduction of baseline cytokine secretion following BAY 11-7082 treatment (^†^< 0.0001, ^‡^< 0.001, ^Δ^< 0.01, ^◊^< 0.05 by 2-way ANOVA with *n* = 4 per condition). Data presented as individual recordings normalized to average baseline measurement per EHT. Significance calculated based on mean value per EHT.

Because innate immune activation is characterized by excess NF-κB signaling, we tested the NF-κB inhibitor BAY 11-7082 since this treatment has previously been shown to improve cardiac outcomes in a desmoglein-2 (*Dsg2*) mouse model and hiPSC-CM model of *PKP2*-related arrhythmogenic cardiomyopathy (27). When treated with NF-κB inhibitor BAY 11-7082, *DSP*tv EHTs p.R1951X and p.E1597X improved contractile function while healthy control EHTs demonstrated no change in fractional shortening (Figure 6A). Furthermore, the impairment of contractile reserve seen in *DSP* p.E1597X was reversed by BAY 11-7082 treatment (Figure 6, B and C). This improvement in contractile function induced by BAY 11-7082 treatment was associated with a reduction in the media cytokine profile compared with vehicle-treated *DSP* p.E1597X (Figure 6, D and E).

We also tested JSH-23, an inhibitor of NF-κB transcriptional activity (46). Consistent with the BAY 11-7082 results, we found that JSH-23 blocked the increase in HMGB1-induced FPD lengthening in hiPSC-CMs in culture (Supplemental Figure 8B). We also tested oridonin because it inhibits the NLRP3 inflammasome, a protein complex implicated in innate immune activation (47). We found that treatment with oridonin plus HMGB1 resulted in a shortened FPD (Supplemental Figure 8B). We found that JSH-23 improved contractile dysfunction of *DSP*tv patient-derived EHTs, but we did not see this same effect with oridonin, suggesting the NF-κB inhibition is important for both contractile dysfunction and arrhythmia propensity (Supplemental Figure 8C).

### Colchicine treatment of EHTs reverses contractile defects in patient-derived EHTs.

**Figure 7:**
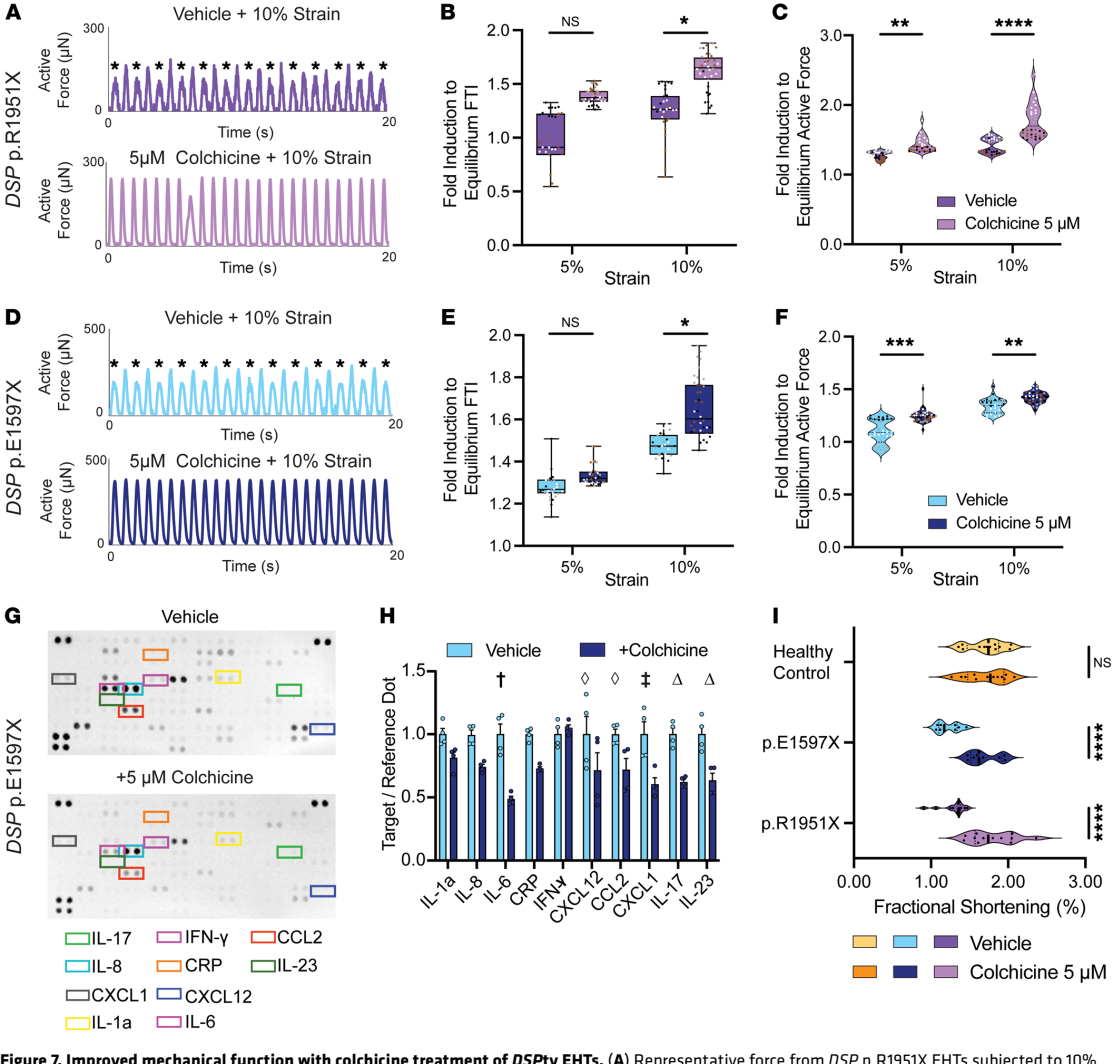
Improved mechanical function with colchicine treatment of *DSP*tv EHTs. (**A**) Representative force from *DSP* p.R1951X EHTs subjected to 10% strain with and without colchicine. Exposure to 10% strain produced marked mechanical alternans (marked with asterisks), which was corrected with colchicine treatment. (**B** and **C**) Colchicine significantly improved relative mechanical force production in *DSP* p.R1951X EHTs at baseline conditions and after isometric strain (*< 0.05, **< 0.01, ****< 0.0001 by 2-way ANOVA, *n* = 3–4 EHTs per condition, labeled as n1 = black dots, n2 = white dots, n3 = gray dots, n4 = brown dots). Box plots show the interquartile range, median (line), and minimum and maximum (whiskers). (**D**) *DSP* p.E1597X EHTs also showed marked alternans (marked with asterisks) after 10% strain, which was improved by colchicine. (**E** and **F**) Colchicine improved relative force in *DSP* p.E1597X EHTs at baseline and with 5% or 10% strain (*< 0.05, **< 0.01, ***< 0.001 by 2-way ANOVA, *n* = 3–4 EHTs per condition, labeled as n1 = black dots, n2 = white dots, n3 = gray dots, n4 = brown dots). (**G** and **H**) Cytokine arrays from p.E1597X EHT media showed a significant reduction of baseline cytokine secretion following colchicine treatment (^†^< 0.0001, ^‡^< 0.001, ^Δ^< 0.01, ^◊^< 0.05 by 2-way ANOVA with *n* = 4 per condition). (**I**) Fractional shortening of *DSP* p.E1597X and p.R1951X EHTs treated with 5 μM colchicine for 48 hours demonstrated improved contractility compared with vehicle control (****< 0.0001 by 2-way ANOVA, *n* = 9–12 EHTs per condition). Data presented as individual recordings normalized to average baseline measurement per EHT. Significance calculated based on mean value per EHT.

The patient with the *DSP* p.E1597X truncation variant was treated with colchicine, with improvement of symptoms since colchicine has been used to treat a broad array of cardiovascular conditions associated with inflammation (48). Applying 10% strain to *DSP* heterozygous EHTs resulted in marked alternans in patient-derived, heterozygous *DSP*tv EHTs (Figure 7), and this contractile reserve deficit was corrected by 5 μM colchicine (Figure 7, A–C, for *DSP* p.R1951X and Figure 7, D–F, for *DSP* p.E1597X). Not only did 5 μM colchicine minimize strain-induced alternans, but also there was an increase in relative force production in response to strain in *DSP* p.R1951X and in *DSP* p.E1597X EHTs (Figure 7, A–F). Colchicine treatment was also associated with a reduction of IL-6, IL-17, and IL-23 release into the media (Figure 7, G and H). This antiinflammatory effect translated into improved fractional shortening at baseline in heterozygous *DSP*tv patient-derived EHTs, and this response to colchicine was not seen in healthy control EHTs (Figure 7I) that did not have reduced contractile reserve.

### Genetic correction of pathogenic DSP variant reduces inflammation.

**Figure 8:**
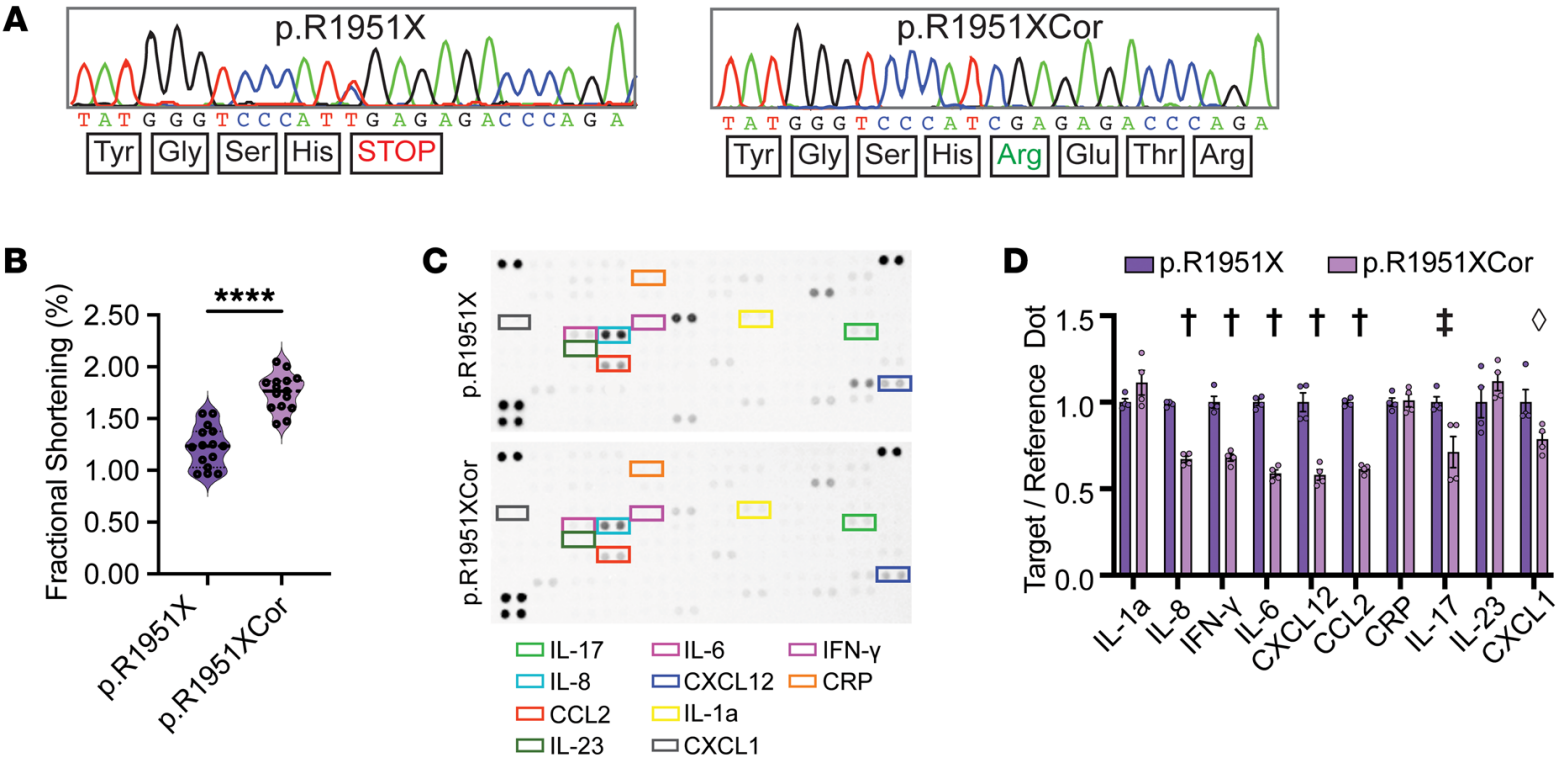
Base editing to correct *DSP* p.R1951X reduces inflammation in EHTs. (**A**) Sanger sequencing of p.R1951X hiPSCs before and after base editing to correct the pathogenic *DSP* variant using gRNA8 and an adenine base editor (ABE). Base editing restored the reading frame, and the corrected line was termed p.R1951XCor. (**B**) Fractional shortening (FS) measurements of p.R1951X and p.R1951XCor EHTs showed improved contractility following genomic correction (****< 0.0001 by 2-tailed *t* test with Welch’s correction; *n* = 15 per condition). (**C** and **D**) Cytokine arrays of EHT media demonstrate significant reduction of cytokine secretion following genomic correction of *DSP* p.R1951X. Targets displayed in colored boxes are quantified in relation to Reference Dots (**D**) (^†^< 0.0001, ^‡^< 0.001, ^◊^< 0.05 by 2-way ANOVA with *n* = 4 per condition). Data presented as mean ± SEM.

The *DSP* p.R1951X pathogenic variant was correctable by base editing (Supplemental Figure 9, A–E). A specific guide RNA, gRNA8, was successful in producing an hiPSC clone with genomic correction of the pathogenic C>T variant in the *DSP* p.R1951X line (Figure 8A), which restored the proper reading frame with minimal bystander effects (Supplemental Table 6). The *DSP* transcript was increased by genetic correction with a concomitant decrease in *NFKB* mRNA (Supplemental Figure 10, A and B). EHTs derived from the corrected hiPSC clone had improved fractional shortening compared with uncorrected *DSP* p.R1951X (Figure 8B). Improved mechanical function was accompanied by increased DPI protein level in p.R1951XCor EHTs (Supplemental Figure 10, C and D) and improved contractile reserve (Supplemental Figure 10E). To assess whether genomic correction influenced the innate immune activation phenotype, cytokine array analysis of EHTs derived from the base-edited, corrected line (*DSP* p.R1951XCor) had normalization of many, but not all, of the cytokines produced in the media (Figure 8, C and D). Interestingly, IL-1α, C-reactive protein, and IL-23 were still elevated in the media of p.R1951XCor EHTs (Figure 8D). Thus, base editing to correct *DSP* p.R1951X EHTs only partly corrected the NF-κB pro-inflammatory cytokines, suggesting contribution of non-*DSP*-induced inflammatory mediators. We further assessed whether p.R1951XCor EHTs had reduced response to TLR stimulation by HMGB1 measured as IL-17 secretion. Genetically corrected p.R1951X EHTs secreted less IL-17 in response to HMGB1 treatment than uncorrected (Supplemental Figure 10F).

We also sought to understand the role of microtubules in mediating antiinflammatory therapy by treating *DSP* p.R1951X EHTs with BAY 11-7082, colchicine, and nocodazole, a microtubule inhibitor that is not known to have antiinflammatory effects (49, 50). Gene expression analysis verified suppression of inflammatory genes by colchicine and BAY 11-7082, with no significant reduction in inflammatory transcripts after nocodazole treatment (Supplemental Figure 10, G–J). We also found the improvement in fractional shortening observed in *DSP* p.R1951X EHTs in response to colchicine therapy was not observed in corrected EHTs or in response to nocodazole treatment (Supplemental Figure 10K), underscoring the role of innate immune activation in driving contractile dysfunction.

## Discussion

### DSP EHTs model the cardiomyocyte aspects of genetically mediated myocarditis.

EHTs are generated by mixing hiPSC-CMs with healthy cardiac fibroblasts at a 9:1 ratio (CMs/cardiac fibroblast) into a fibrin or collagen matrix, then maturing for at least 20 days (35, 36). This EHT 3D scaffold allows cardiomyocytes to align and form intercellular connections. Unlike the intact heart, EHTs are not supported by a circulatory system, nor do they include circulating or tissue-resident lymphocytes and macrophages, which are well documented to be present in myocarditis, including *DSP*-related myocarditis (51, 52). Overall, the mechanical deficits in EHTs were more profound in homozygous *DSP*^–/–^ EHTs, and these findings mirror what is seen clinically where *DSP* heterozygous truncations are more likely to have more normal left ventricular function in the setting of genetic myocarditis compared with *TTN* heterozygous truncations (4). Both heterozygous and homozygous *DSP* EHTs displayed elevated baseline innate immune activation, and additionally these EHTs had an excessive response to TLR agonists. These observations are highly consistent with the clinical presentation of patients with *DSP*tv, in which a concealed phase of normal left ventricular function precedes overt structural changes or arrhythmias. With additional stimuli, the concealed phase can transition to a “hot phase” of myocarditis, characterized by inflammation and accompanying arrhythmias (9, 53, 54). The cytokine release is expected to attract lymphocytes and other inflammatory cells, further exacerbating the hot phase.

*DSP* EHTs had a sterile inflammation profile with strong upregulation of innate immune pathways regulated by NF-κB, like IL-17 and IL-23. Cytokines IL-17 and IL-23 are known to contribute to tissue-specific inflammatory pathologies associated with desmosome-related skin disease (55). Base editing to correct the pathogenic allele *DSP* p.R1951X reduced IL-17 but not IL-23 secretion. IL-17 is therefore likely to be a critical driver of *DSP* pathology and represents an attractive candidate for therapy. *DSP*-driven skin findings have been treated with ustekinumab, an antibody directed at the p40 subunit of IL-23 (56). We tested the NF-κB inhibitor BAY 11-7082 since this has been shown to be useful in a mouse *Dsg2* model of arrhythmogenic cardiomyopathy (27, 28), and we found that this agent improved the depressed contractility in *DSP* EHTs. Cardiac *Dsp* deletion in the mouse produced gene expression shifts in cell death pathways, including pyroptosis, apoptosis, and necroptosis (collectively called PANoptosis) (33). Curiously, we found no evidence of the involvement of these pathways in the *DSP* EHT models presented herein, with cell survival rates similar across the hiPSC-CM differentiation and EHT fabrication process. The PANoptosis signature may reflect differences in mouse physiology or be driven by cell types absent from this EHT model. TF motif enrichment analysis of differential gene expression in *DSP^–/–^* tissues did not identify ZBP1, a master regulator of PANoptosis; however, motifs for other stress markers like C/EBP were observed.

We found that colchicine, a broad antiinflammatory agent, reversed innate immunity–stimulated mechanical and electrical defects in *DSP* EHTs, and we studied this agent because it had been used clinically in the patient presenting with myocarditis. Colchicine has been shown to be useful in reducing atrial fibrillation after cardiac surgery or catheter ablation and additionally has been shown to reduce adverse cardiac outcomes in other settings (57). Given the range of cytokines released by *DSP*-deficient EHTs, the patient’s improvement after colchicine is consistent with its broadly antiinflammatory activity and its dual role as a microtubule and NF-κB inhibitor. Since nocodazole did not elicit similar effects and is a microtubule-targeted agent, it is possible that colchicine is more potent or targets a specific interaction with NF-κB in a manner not replicated by nocodazole. Additional clinical studies are needed to evaluate the efficacy of colchicine for genetically mediated myocarditis. We also found that genetic correction of a specific *DSP* pathogenic variant did not fully correct all aspects of cytokine release, suggesting that there are other genetic contributors to innate immune activation in cardiomyocytes. It is also possible that epigenetic changes persist within the hiPSCs themselves that persist even after gene editing.

### Mechanical and electrical defects in DSP EHTs.

Bliley and colleagues demonstrated altered contractility in patient-derived *DSP* EHTs under conditions of diastolic lengthening that increased desmosomal gene and protein expression (39). In comparing gene expression signatures between *DSP^+/+^* and *DSP^–/–^* EHTs, we observed a failure to upregulate intermediate filament organizational genes, consistent with defective intercellular connectivity in *DSP-*deficient cardiomyocytes. *DSP^–/–^* EHTs not only increased inflammatory protein genes, but we also observed upregulation of *PIEZO1*, a mechanosensitive channel gene. Desmosomes and desmoplakin contribute to a mechanically strong link in epithelial cells and cardiomyocytes, so the upregulation of additional mechanically responsive genes may be particularly relevant. In macrophages, PIEZO1 is required for inflammatory signaling, so it is possible that similar pathways are activated with desmosomal disruption in cardiomyocytes and skin (58, 59). In vivo, this disrupted barrier may permit or enhance inflammatory cell infiltrate or even excessively recruit inflammatory cells through the secretion of lymphokines. Importantly, paracrine signaling from cardiomyocytes can amplify inflammation through local effects and by recruiting leukocytes to the myocardium, further intensifying mechanical defects (60, 61). The cytokines we observed secreted from *DSP* EHTs are positioned to recruit circulating myeloid cells or activate tissue-resident immune cells.

Pérez-Hernández et al. selectively deleted *Pkp2* in vivo in mouse cardiomyocytes, demonstrating upregulation of many pathways associated with viral and bacterial infection gene expression (29), similar to some of the pathways we observed in *DSP* EHTs and rat NRVMs. In the clinical setting, *PKP2* mutations target the RV, leading to arrhythmias and fibrofatty right ventricular replacement. Although the term arrhythmogenic cardiomyopathy encompasses desmosomal gene mutations as well as other cardiomyopathy genes, the selective targeting of right versus left ventricles is not well explained, nor is it well replicated in mouse models. Similarly, human EHTs cannot distinguish between right and left ventricular processes, and more advanced organoids are needed for this.

### Study limitations and conclusions.

The data presented here demonstrate baseline elevation of NF-κB signaling and innate immune activation in *DSP* EHTs but also reveal a heightened sensitivity to additional TLR signaling, creating a model for the cardiomyocyte aspects of myocarditis. This organoid system lacks the additional cell types that would further exacerbate myocarditis pathology, yet at the same time, these studies highlight the role of the cardiomyocyte itself in genetically mediated myocarditis. Furthermore, human EHTs represent an immature cardiomyocyte, distinct from the mature cells of the human myocardium. The EHTs used in these studies contain *DSP*-mutant cardiomyocytes, and in this system, the cardiac fibroblasts were derived from healthy controls. Many different noncardiomyocyte cell types contribute substantially to myocarditis by both accelerating pathology and mediating its resolution. Although these studies used *DSP* disruption as its genetic model of myocarditis, many other genes have been implicated in this process, including *TTN*, *LMNA*, *FLNC*, *PKP2*, and others. More studies are needed to demonstrate whether sustained and stimulated innate immune activation is similar across these different genetic models and to establish the similar and dissimilar features to ARVC.

## Methods

### Sex as a biological variable.

Chromosomal sex of cell lines used in this study may be found in Supplemental Tables 1 and 2. Control cell lines were sex matched to account for any potential variability in EHT models.

### Generation and maintenance of hiPSC lines.

HiPSC lines were generated from PBMCs isolated by combining equal volumes serum with Histopaque-1077 Cell Separation Media (MilliporeSigma, catalog 10771) and filtering with 12 mL Lucosep separation tubes (Greiner Bio-one, catalog 163290P) according to manufacturer protocols. Isolated PBMCs were reprogrammed using the CytoTune-iPS 2.0 Sendai Reprogramming Kit (Invitrogen, catalog A16517) according to manufacturer protocol. *DSP^+/+^* and *DSP^–/–^* cell lines were generated from human skin fibroblasts (Coriell, sample GM03348) that were reprogrammed using electroporation with pCXLE-hOCT3/4-shp53-F (Addgene plasmid 27077), pCXLE-hSK (Addgene plasmid 27078), and pCXLE-hUL (Addgene plasmid 27080) as described previously (62). Successful reprogramming was assessed via cell morphology, ability to differentiate, and purity as measured with SSEA3 staining (BD, catalog 560879) via flow cytometry (63). HiPSCs were maintained in mTeSR-1 (STEMCELL Technologies, catalog 85850) and mTeSR-Plus (STEMCELL Technologies, 100-0276) on Matrigel-coated, 6-well plates (Corning, catalog 354230). Cells were passaged once every 4–5 days at 80%–90% confluence by washing with 1× PBS and incubating with ReLeSR (STEMCELL Technologies, catalog 100-0483) for 3 minutes at 37°C.

hiPSC-CMs were differentiated using small molecule modulation of the Wnt signaling pathway (64). Briefly, when cells reached approximately 95% confluence, hiPSCs were treated for 24 hours with 6 μM CHIR99021 (Tocris, catalog 5148) in CDM3 (RPMI 1640, catalog 11875-119) with l-glutamine, 213 mg/mL l-ascorbic acid 2-phosphate, and 500 mg/mL recombinant human albumin (MilliporeSigma, catalog A1653) (64). Cells were allowed to recover for 24 hours in RPMI-CDM3. On day 2 of differentiation, cells were treated with 2 μM Wnt-C59 (Tocris, catalog 5148) in RPMI-CDM3 for 48 hours. RPMI-CDM3 was changed every 2 days until cells matured into beating monolayers. On days 8–12 of differentiation, wells with beating cardiomyocytes were dissociated with 1 mg/mL collagenase IV (Worthington Biochemical Corporation, catalog LS004188) in HBSS, (Thermo Fisher Scientific, catalog 14025092) with 10 mM HEPES, 2 μM Thiazovivin (STEMCELL Technologies, catalog 72254), and 30 μM *N*-Benzyl-*p*-Toluenesulfonamide (TCI, catalog B3082) for 2 hours at 37°C. Single hiPSC-CMs were filtered through a 100 μm cell strainer (Fisherbrand, catalog 22-363-549) and purified via PSC-Derived Cardiomyocyte Isolation Kit, human (Miltenyi Biotec, catalog 130-110-188). Briefly, hiPSC-CMs were labeled with magnetic bead–conjugated antibodies in a Non-Cardiomyocyte Depletion Cocktail and purified by negative selection via magnetic column. HiPSC-CMs were counted using Nexcelom Bioscience Cellometer Acridine Orange/Propidium Iodide cell viability assay according to manufacturer protocol. For each differentiation, 2 × 10^6^ hiPSC-CMs were fixed in 4% paraformaldehyde and stained with anti–cardiac troponin T (BD, catalog 565744). Cardiomyocyte purity was assessed via flow cytometry as described (65), and only differentiations of more than 90% purity were used.

### Plasmids and gene editing.

*DSP*^–/–^ cells were generated using gRNAs designed by CRISPOR (66), which targeted *DSP* exon 24 (Supplemental Table 7). gRNAs 190 and 202 were placed into the Cas9-expressing pSpCas9(BB)-2A-Puro (PX459) V2.0 (Addgene plasmid 48138) vector (Blue Heron Biotech). Nucleofection with gRNA and Cas9 used 1.5 μg each of pSpCas9(BB)-2A-GFP with gRNA 190 and gRNA 202. pSpCas9 (BB)-2A-GFP–nucleofected cells were allowed to recover for 48 hours in mTesR Plus supplemented with 2 μM Thiazovivin prior to further selection. At 48–96 hours after transfection, colonies were treated with 0.2 μg/mL puromycin (Thermo Fisher Scientific, catalog A1113803) and then returned to daily media exchange with mTesR Plus supplemented with 10% CloneR (STEMCELL Technologies, catalog 5888) and 2 μM Thiazovivin. After 10 days, colonies were isolated and then screened by Sanger sequencing and TIDE analysis (67). Final clones were confirmed using amplicon sequencing (Amplicon EZ sequencing, Azenta Life Sciences).

For base-editing correction of *DSP* c.5851 C > T p.R1951X hiPSC line, gRNAs were designed using BE Hive (68). gRNAs 6, 7, and 8 were ligated into pmCherry_gRNA plasmid (Addgene plasmid 8045) vector. For the base editing, nucleofection reactions contained 1.5 μg of pmCherry_gRNA plasmid with the target gRNA and 4.5 μg of pCMV_ABEmax_P2A_GFP (Addgene plasmid 112101) or pCMV-T7-ABE8e-SpRY-HF1-P2A-EGFP (Addgene plasmid 197507). Nucleofection reactions used the Neon Transfection System (Invitrogen MPK5000S) in 6-well, 100 μL suspension format according to manufacturer protocols. Briefly, 1 hour before nucleofection, hiPSCs at 70%–90% confluence were treated with 2 μM Thiazovivin Rock Inhibitor (MilliporeSigma, catalog SML1045). HiPSCs were digested with TrypLE (Thermo Fisher Scientific, catalog 12563011) and resuspended in Resuspension Buffer at 8 × 10^5^ cells/100 μL (Invitrogen, catalog MPK10096) and incubated with 6 μg of plasmid DNA containing total genome-engineering machinery with gRNA, control DNA, or no DNA. ABE-nucleofected colonies were treated with mTesR Plus supplemented with 100 μg/mL Primocin (InvivoGen catalog ant-pm-05) and 2 μM Thiazovivin for the first 24 hours after nucleofection and then returned to daily media exchange with mTesR Plus. Three days after nucleofection, pmCherry and GFP double-positive cells were collected by fluorescence-activated cell sorting. Genotype of collected colonies was confirmed via Sanger and amplicon sequencing. Editing efficiency was estimated using with EditR (69) to analyze chromatograms resulting from Sanger sequencing. HiPSC clones were expanded to establish the *DSP* p.R1951X > R (gRNA8) hiPSC line.

### Human cardiac fibroblast culture.

Human ventricular cardiac fibroblasts were obtained from Promocell (catalog C-12377) and expanded. Initial cultures were expanded maximally using Fibroblast Growth Medium (Promocell C-23010). Cells were grown until 80%–90% confluent and then isolated using DetachKit (Promocell C-41200) and stored at 2 × 10^6^ cells/mL at –80°C with Freezing Medium Cryo-SFM (Promocell C-29910) according to manufacturer protocol. Frozen stock fibroblasts were then thawed fresh for each EHT batch during hydrogel formation.

### EHT generation.

EHTs were generated in multiwell format in a protocol adapted from Tiburcy et al. (Supplemental Figure 1) (35). Briefly, purified hiPSC-CMs were combined with human cardiac fibroblasts in a 9:1 ratio for a total of 0.55 × 10^6^ total cells per EHT. Cells were suspended in RPMI with B-27 supplement (Thermo Fisher Scientific, catalog 17504044) and combined over ice with 44 μL collagen type I (6.5 mg/mL, MilliporeSigma, catalog 804614), 44 μL of 2× RPMI (Thermo Fisher Scientific, catalog 318000022), and 6 μL of 0.1N NaOH for a total of 198 μL per EHT. A total of 180 μL of resultant hydrogel mixture was pipetted evenly into wells on a 48-well engineered human myocardium plate (myrPlate myriamed) for EHT fabrication, resulting in a final cell density of 500,000 cells/EHT. Following initial casting (day 0), the 48-well plate was incubated at 37°C for 1 hour and then supplemented with EHT media containing DMEM low glucose (MilliporeSigma, catalog D5546), 10% horse serum (heat inactivated, New Zealand origin, Gibco, catalog 26050088), 1% Penicillin/Streptomycin (Thermo Fisher Scientific, catalog 15070063), and 0.1% human insulin (10 mg/mL, MilliporeSigma catalog 19278). For the first 72 hours following EHT generation, medium was supplemented with 5 μg/mL recombinant human TGF-β1 (CHO derived, PeproTech, catalog 100-21C). Day 3 after tissue casting, TGF-β1 was removed from culture medium, and 500 μL per well of EHT medium was exchanged every 2–3 days for tissue maturation prior to downstream applications at days 21–35. For physiological measurements, *DSP*tv and control EHTs were prepared in parallel and similarly processed to control for technical differences from EHT batch to batch.

### Contractility assessment.

EHT contractility was assessed following 3–5 weeks of culture. Fractional shortening was assessed by optical microscopy on the commercial myriamed EHT platform (TM-5 posts, spring constant 1.5 mN/mm with 5 mm post-to-post distance). EHTs were recorded beating spontaneously in EHT culture media using a Zeiss stereomicroscope (catalog 435425-9000-000) modified with a Motic SMZ-171 head and Motic Moticam 1080N BMH camera. A minimum of 3 independent beats per EHT were used for analysis. Post deformation was made blinded to genotype and treatment using ImageJ (NIH) and averaged across 3 beats per EHT to provide fractional shortening data.

For direct force measurements of isometric contractions, EHTs were gently transferred from the culture posts and mounted onto a temperature-controlled muscle force characterization apparatus (Aurora Scientific, 801C-1900, equipped with a 400B 50 mN force transducer, 322D high speed length controller, and 701C stimulator, mounted on a Nikon Eclipse Ti2 inverted microscope). Measurements were made at 75 beats per minute, stimulated at approximately 30 mA at 37°C in Tyrode’s solution (140 mM NaCl, 5.4 mM KCl, 1.43 mM CaCl_2_, 1 mM MgCl_2_, HEPES 25 mM, and 10 mM d-glucose at pH 7.3). For assessment of drug treatment on contractility, EHT media was exchanged with specified drug concentrations or vehicle control 48 hours before measurements. For LPS and HMGB1 experiments, EHTs were cultured in serum-free media (RPMI with B-27 supplement) for 48 hours before measurement; the absence of serum may lead to altered force measurements.

### Optical mapping.

For electrophysiology measurements, dual-parameter optical mapping was applied to EHTs to acquire transmembrane potential and intracellular calcium signals. The setup of the mapping system was described in detail in previous studies (70, 71). Briefly, EHTs were submerged in oxygenated and recirculated Tyrode’s solution (140 mM NaCl, 4.5 mM KCl, 1.8 mM CaCl_2_, 1 mM MgCl_2_, 10 mM HEPES, 10 mM glucose) supplemented by 15 μM blebbistatin (Cayman Chemical 13186) at physiological pH (7.4) and temperature (37°C). After a 10-minute equilibration period, the perfusate circulation was stopped, and extra perfusate in the bath was removed, followed by each EHT being incubated with 150–200 μL RH237 (30 μL of 1.25 mg/mL dye + 970 μL perfusate, Biotium 61018) for 1 minute. The circulation was then resumed to wash the excess dye for 1 minute. Similarly, the EHTs were stained with Rhod2-AM (30 μL of 1 mg/mL dye + 30 μL Pluronic F-127 + 940 μL perfusate, Thermo Fisher Scientific R1244, Biotium 59005).

Electrical stimulation was applied by placing 2 platinum plates on either side of the EHT, and the voltage threshold of capture was determined. Restitution protocol was applied by pacing the EHTs at basic cycle length starting from 2 seconds and increasing the pacing rate until loss of 1:1 capture. At each cycle length, the tissue was illuminated by 520 nm excitation light to emit 590 nm (Rhod2-AM) and 690 nm (RH237) light, which was captured by 2 CMOS cameras (MiCAM05, SciMedia) with 100 × 100 pixel resolution at 1,000 frames per second. The duration of each recording was 5 seconds. BV Workbench 2.6.1 (SciMedia) was used for camera alignment and acquisition. The mapping data analysis was performed in Rhythm 3.0 (https://github.com/optocardiography/Rhythm-3.0; commit ID 68534a4). Transmembrane potential and calcium signals were used to calculate APD, CV, and CaTD. APD_80_ and CaTD_80_ were calculated by measuring the time interval between the initiation of an upstroke and 80% of the repolarization or calcium transient decay. CV was calculated by determining the time taken to travel a known distance on an EHT surface in a specified direction (transverse or longitudinal).

### Cytokine array and ELISA.

Protein measurements from EHT media were performed with the Proteome Profiler Human XL Cytokine Array Kit (R&D Systems, catalog ARY022B) and Human IL-1 beta/IL-1F2 Quantikine HS ELISA Kit (R&D Systems, catalog HSLB00D) and Human IL-17 Quantikine HS ELISA Kit (R&D Systems, catalog HS170) according to manufacturer protocols. Briefly, 500 mL of EHT media was collected 48 hours after media exchange with standard media or specified drug treatments and controls. Fresh samples were then diluted with appropriate reagents and used as input for cytokine measurements.

### Multielectrode array measurements.

Purified hiPSC-CMs (2 × 10^4^ hiPSC-CMs per well) were replated onto a 96-well CardioExcyte Standard Stim MEA plate (Nanion Technologies, catalog 201003) and allowed to recover for 7–10 days. Media containing treatment compounds were administered 48 hours and 1 hour prior to plate reading on the CardioExcyte 96 platform (Nanion Technologies) at specified concentrations and with vehicle controls (Supplemental Table 8). Fully confluent, beating hiPSC-CM monolayers were paced with sine StimMode protocol at 1 Hz with 5 ms burst length, 1 kHz burst frequency, and 20% intensity. Sweep durations lasted 30 seconds with a repetition interval of 10 minutes, for a total assay time of 4–5 hours. Quality control and data analysis were performed using DataControl software for the CardioExcyte Nanion.

### Immunoblotting.

HiPSC-CMs from monolayer cultures were washed with cold 1× PBS and collected in cell lysis buffer with protease (MilliporeSigma, catalog 11836170001) and phosphatase inhibitor (MilliporeSigma, catalog 4906837001) cocktails using a cell scraper. Supernatant was collected after samples were centrifuged at 2,500*g* for 5 minutes at 4°C. Pierce Protein BCA kit was used to quantify total protein in samples according to manufacturer protocols (Thermo Fisher Scientific, catalog 23227). Samples were prepared and stored using 4× Laemmli protein sample buffer (Bio-Rad, catalog 1610747). Samples were warmed for 2 minutes at 99°C and separated using a 5%–15% precast protein gel (Bio-Rad, catalog 4561083) at 120 V for 2 hours. Transfer to PVDF membrane (Bio-Rad, catalog 1620177) was for 3 hours at 900 mA at 4°C. PVDF membranes were blocked with T20 blocking buffer (Thermo Fisher Scientific, catalog 37543) for 1 hour at room temperature and incubated overnight at 4°C with primary antibodies as listed in Supplemental Table 9. Immunoblots were washed with 1× TBS-Tween and secondary antibodies goat anti-mouse (Jackson ImmunoResearch, catalog 115-035-003, 1:2,500) and goat anti-rabbit (Jackson ImmunoResearch, catalog 111-035-003, 1:2,500) for 1 hour at room temperature. Immunoblots were imaged using PICO (Thermo Fisher Scientific, catalog 34580) chemiluminescence substrates on the iBright 1500 (Invitrogen). Protein loading was assessed using Memcode reversible protein stain kit (Thermo Fisher Scientific, catalog 24585) according to manufacturer protocols. Blot quantification was performed using ImageJ.

### Immunofluorescence microscopy.

For microscopy of hiPSC-CMs in monolayers, hiPSC-CMs (3 × 10^5^ cells) were replated on Matrigel-coated 12 mm Micro coverglass slips (Electron Microscopy Sciences, catalog 72231-01) in a 24-well format, and 7–14 days later, fully confluent, beating hiPSC-CM monolayers were fixed on coverglass slips with 2% paraformaldehyde. For microscopy of 3D tissues, EHTs (days 21–30) were fixed in 2% methanol for 30 minutes 4°C and equilibrated to 30% sucrose in PBS prior to mounting in OCT (Thermo Fisher Scientific catalog 14-373-65). Cryosectioning was performed at chamber temperature of –15°C and sectioning head at –20°C using Leica Biosystems CM1860 UV. Samples were sectioned at 10 μm and mounted directly onto slides. Fixed samples were washed with PBS, permeabilized with 0.2% Triton, and blocked with 5% bovine serum albumin (MilliporeSigma, catalog A3294) for 1 hour at room temperature. Samples were incubated with primary antibodies in 5% BSA overnight at 4°C. Slides were washed with 0.1% Tween, and secondary antibodies were incubated in 5% BSA for 1 hour at room temperature. Samples were imaged using Keyence BZ-X810. Florescence quantification was performed using ImageJ, and colocalization measurements were performed on images using BZ Analyzer (Keyence).

### RNAi-mediated knockdown.

Oligonucleotide treatment in NRVMs was performed as previously reported (26, 30). Oligonucleotide sequences targeting genes of interest can be found in Supplemental Table 10.

### RNA isolation.

RNA was isolated from EHTs using TRIzol Reagent (Thermo Fisher Scientific). Each sample contained 2 EHTs from the same batch dissolved in 1,000 μL TRIzol. The entire sample was added to a tube containing 250 μL of silica-zirconium beads. Following homogenization for 60 seconds in Mini-BeadBeater (BioSpec Products), samples were cooled on ice for 1 minute and then allowed to recover at room temperature for 5 minutes. Lysates were centrifuged at 12,000*g* for 5 minutes, and collected supernatant was combined with 200 μL chloroform and incubated at room temperature for 10 minutes with periodic vigorous shaking. Lysates were centrifuged at 4°C and 12,000*g* for 15 minutes, and aqueous-phase reactant was collected and combined with 600 μL 70% ethanol. This final reaction product was then used as starting material for RNA isolation using input to the Aurum Total RNA Mini Kit (Bio-Rad) according to manufacturer specifications. cDNA libraries were synthesized from isolated RNA using qScript cDNA SuperMix (QuantaBio) and used for downstream applications. qPCR analysis was performed as previously described (65) with the ∆∆Ct method using the geometric mean expression of cardiomyocyte reference genes and primers listed in Supplemental Table 7.

### RNA-Seq analysis.

RNA-Seq reads were analyzed as previously described (62). Briefly, raw reads were trimmed with trimmomatic (v0.36) and aligned to the human genome (hg19) using STAR with default settings. Uniquely aligned reads were assigned to genes using htseq-count. Raw counts were normalized with EdgeR. DEGs were defined as any gene with an FDR-corrected *P* < 0.05. ggPlot2 was used to graph the log-normalized and depth-normalized gene expression values generated by RNA-Seq. Heatmaps were visualized from *z* scores with medians in RStudio. Significantly downregulated and upregulated genes were separated based on the sign of their log fold-change value. Ensembl gene IDs were inputted into the Metascape online tool for enrichment of GO terms (72). Motif enrichment analysis of DEGs was performed using HOMER (73).

### Statistics.

Data were analyzed using GraphPad Prism, v10, and the specific statistical tests were selected based on normality of data distribution, experimental design, and number of comparisons. *P* < 0.05 was considered statistically significant.

### Study approval.

All participants provided written informed consent for cell donation and access to medical record information under the approval of Northwestern University Institutional Review Board (STU00104548). Probands had clinical next-generation-based cardiomyopathy panel sequencing as part of standard of care, and specific pathogenic variants were confirmed via Sanger sequencing in the derived cell lines. NRVMs were isolated with approval of the Northwestern University Institutional Animal Care and Use Committee, and all animal care protocols conformed to the *Guide for the Care and Use of Laboratory Animals* (National Academies Press, 2011).

### Data availability.

All supporting data are available within the article and its Supporting Data Values file. RNA-Seq data generated in this study have been deposited in the National Center for Biotechnology Gene Expression Omnibus database under accession code GSE248187.

## Author contributions

DFS, KJG, and EMM conceived and designed the project. DFS, DEF, IAC, BL, ADD, JO, TOM, GT, CH, and SAG performed and analyzed experiments. DFS and MB developed hiPSC cell lines. DEF, LDC, PWB, ALG, ARD, MJP, IRE, KJG, and EMM provided critical resources. DFS and EMM drafted the manuscript. DEF, TOM, and KJG critically revised the manuscript.

## Supplementary Material

Supplemental data

Unedited blot and gel images

Supporting data values

## Version 1 05/20/2024

In-Press Preview

## Version 2 07/01/2024

Electronic publication
