# Supplementary material for: Susceptibility to innate immune activation in genetically mediated myocarditis
Source: J Clin Invest. 2024 May 16;134(13):e180254. doi: 10.1172/JCI180254 (PMC11213508; doi:10.1172/JCI180254)
Supplement: Supplemental data [file jci-134-180254-s251.pdf]

SUPPLEMENTARY FIGURES AND TABLES FOR SELGRADE ET AL.

Supplementary Table 1. Patient-derived hiPSC lines used

***DSP* genotype**	**Sex**	**Clinical Presentation**	**Abbreviation**
WT/WT	XX	N/A	Healthy Control
c.4789 G>T	XX	Nonsustained ventricular tachycardia (NSVT), age 22	p.E1597X
c.5851 C>T	XX	Congestive heart failure, age 44	p.R1951X

Supplementary Table 2. Gene-edited hiPSC line

***DSP* Allele 1**	***DSP* Allele 2**	**Sex**	**Abbreviation**
WT	WT	XY	*DSP*$^{+/+}$
c.5852del13 p.R1951fs*16	c.5856del2 p.E1952fs*3	XY	*DSP*$^{-/-}$

Supplementary Table 3. Off target analysis of CRISPR-Cas9 Deletion of *DSP*

Guide	Location	Annotation	Off Target Editing
DSPtv_202	Chr14:87151259-87151345	Intergenic (RP11-736P16.1|RP11-1141N12.1)	Negative
DSPtv_190	Chr10:65724401-65724472	Intergenic (AC022538.1|RP11-222A11.1)	Negative
DSPtv_190	Chr4:10088168-10088253	Intron (WDR1)	Negative
DSPtv_202	Chr3:99646016-99646078	Intron (COL8A1)	Negative

Supplementary Table 4. Fractional Shortening (%)

EHT	Standard Culture Media
DSP$^{+/+}$	0.90 ± 0.14
DSP$^{-/-}$	0.44 ± 0.20
Healthy Control	2.10 ± 0.29
p.R1951X	1.63 ± 0.37
p.E1597X	1.58 ± 0.34

Supplementary Table 5. Fractional Shortening (%)

EHT	Control Media	+LPS	+HMGB1
DSP$^{+/+}$	1.33 ± 0.34	0.38 ± 0.15	0.60 ± 0.25
DSP$^{-/-}$	0.63 ± 0.17	0.26 ± 0.08	0.35 ± 0.12
Healthy Control	1.80 ± 0.30	0.70 ± 0.20	0.80 ± 0.04
p.R1951X	1.50 ± 0.30	0.33 ± 0.02	0.52 ± 0.05
p.E1597X	1.60 ± 0.20	0.50 ± 0.03	0.61 ± 0.04

Supplementary Table 6. Off target analysis of Adenine Base Editing (gRNA8)

Location	Annotation	Off Target % A to G						
chr1: 39918463-39918485	Exon (MACF1)	**A_8**	**A_{12}**	**A_{14}**	**A_{18}**	**A_{20}**		
		0.05%	0.21%	0.16%	0.05%	0.04%		
chr17: 10232878-10232900	Intron (MYH13)	**A_8**	**A_9**	**A_{14}**	**A_{16}**	**A_{18}**	**A_{20}**	
		0.16%	0.14%	0.27%	0.19%	0.12%	0.10%	
chr9: 134079491-134079513	Intron (NUP214)	**A_8**	**A_9**	**A_{12}**	**A_{14}**	**A_{16}**	**A_{18}**	**A_{20}**
		0.01%	0.01%	0.30%	0.32%	0.21%	0.01%	0.01%
chr9: 14675169-14675191	Intron (ZDHHC21)	**A_1**	**A_8**	**A_9**	**A_{12}**	**A_{14}**	**A_{16}**	**A_{20}**
		0.15%	0.08%	0.07%	0.23%	0.25%	0.01%	0.06%

Supplementary Table 7. qPCR Primers and Cas9 Guides

Name	Sequences	Notes
DSPtv_202 sgRNA	ACTCAGTCTGGGTCTCTCGA	PAM: -TGG
DSPtv_190 sgRNA	CGGTCCACTCACACTCAGTC	PAM: -TGG
1951_ABE_sgRNA8	GTCTCTCAATGGGACCCATA	PAM: -TGG
1951_ABE_sgRNA7	TCTCTCAATGGGACCCATAT	PAM: -GGG
1951_ABE_sgRNA6	CTCTCAATGGGACCCATATG	PAM: -GGC
DSP ex 23-24 qPCR	F. AGCGCCTGGAGTGTGAGAA R. ATAGCCTCCTCCTTGCGGG	Efficiency = 1.00
NFKB qPCR	F. CCAGGAGGCCGAACGC R. GGTATGGGCCATCTGCTGTT	Efficiency = 0.99
IL1B qPCR	F. CAGGCTGCTCTGGGATTCTC R. AGCCATCATTTCACTGGCGA	Efficiency = 0.95
IL1A qPCR	F. ACTGATGATGACCTGGAGGC R. AAGGTGCTGACCTAGGCTTG	Efficiency = 1.00
IL6 qPCR	F. TGAACTCCTTCTCCACAAGC R.GGGCGGCTACATCTTTGGAA	Efficiency = 0.94
RELA qPCR	F. GAATGGCTCGTCTGTAGTGC R.GGGCTGCTCAATGATCTCCA	Efficiency = 1.04
IL-8 qPCR	F. CTCTCTTGGCAGCCTTCCTG R.TTGGGGTGGAAAGGTTTGGAG	Efficiency = 1.09
TLR4 qPCR	F. ATGATGCCAGGATGATGTCTG R.AATATTAGGAACCACCTCCACG	Efficiency = 1.07
NEK7 qPCR	F. CCACCTGTTCCTCAGTTCCA R.CTTCACTAAATTGTCCGCGACC	Efficiency = 0.96
AGER qPCR	F. GGAACAGCAGTTGGAGCCT R.CCGGGCTGTGATGTTTTGAG	Efficiency = 0.99
PYC qPCR	F. TCTACCTGGAGACCTACGGC R.CTGAGGAGGGGCCTGGAT	Efficiency = 1.03
TNFa qPCR	F. GAGGCCAAGCCCTGGTATG R. CGGGCCGATTGATCTCAGC	Efficiency = 0.94

Supplementary Table 8. Pharmacological Compounds Used

Compound	Source/ Cat No.	Concentration
BAY 11-7082	Sigma B5556	10 μM
colchicine	Thermofisher Scientific J61072.03	5 μM
HMGB-1	Acrobiosystems HM1H5220100UG	100 ng/mL
JSH-23	Sigma J4455	10 μM
lipopolysaccharide (LPS)	Sigma L4516	1 mg/mL
oridonin	Selleckchem S2335	2 μM
nocodazole	Sigma M1404	30 μM

Supplementary Table 9. Antibodies Used

Target	Clone/ Cat No.	Clonality	Usage
Desmoplakin-I (ROD)	2.17	mAb, mouse	IF
Desmoplakin-I&II (C-terminus)	NW6	pAB, rabbit	WB
Plakoglobin	1408 (Aves Laboratories)	mAB, chicken	IF
MYBPC3	sc-137180 (Santa-Cruz)	mAB, mouse	WB
PKP2	651101 (Progen)	mAB, mouse	WB
Connexin-43	C6219 (Sigma)	mAB, rabbit	WB, IF
SSEA-3	560879 (BD Biosciences)	mAb, rat	Flow
TNNT2, cardiac	565744 (BD Biosciences)	mAb, mouse	Flow
β-Tubulin	66240 (Proteintech)	mAB, mouse	WB

Supplementary Table 10. siRNA Targeting Sequences used in NRVMs

Target	Sequence (5′ to 3′)
Dsp	AAACCGGAAACATCATCTCTT
Dsp	TGGTAATAGTTGACCCAGAAA
Jup	TGTTCAATAAGGTTCATCACC
Jup	TACAATGGCCGACTTGAGTAG
Jup	TAGAGCAGCAGGTTGTGCAGT
Jup	TGGTTGAGCAGTTTCACAATG
Pkp2	TGTGAATATGCT- GGATGCAGA
Pkp2	TGGAACTTGTCCTCTAGTGAT

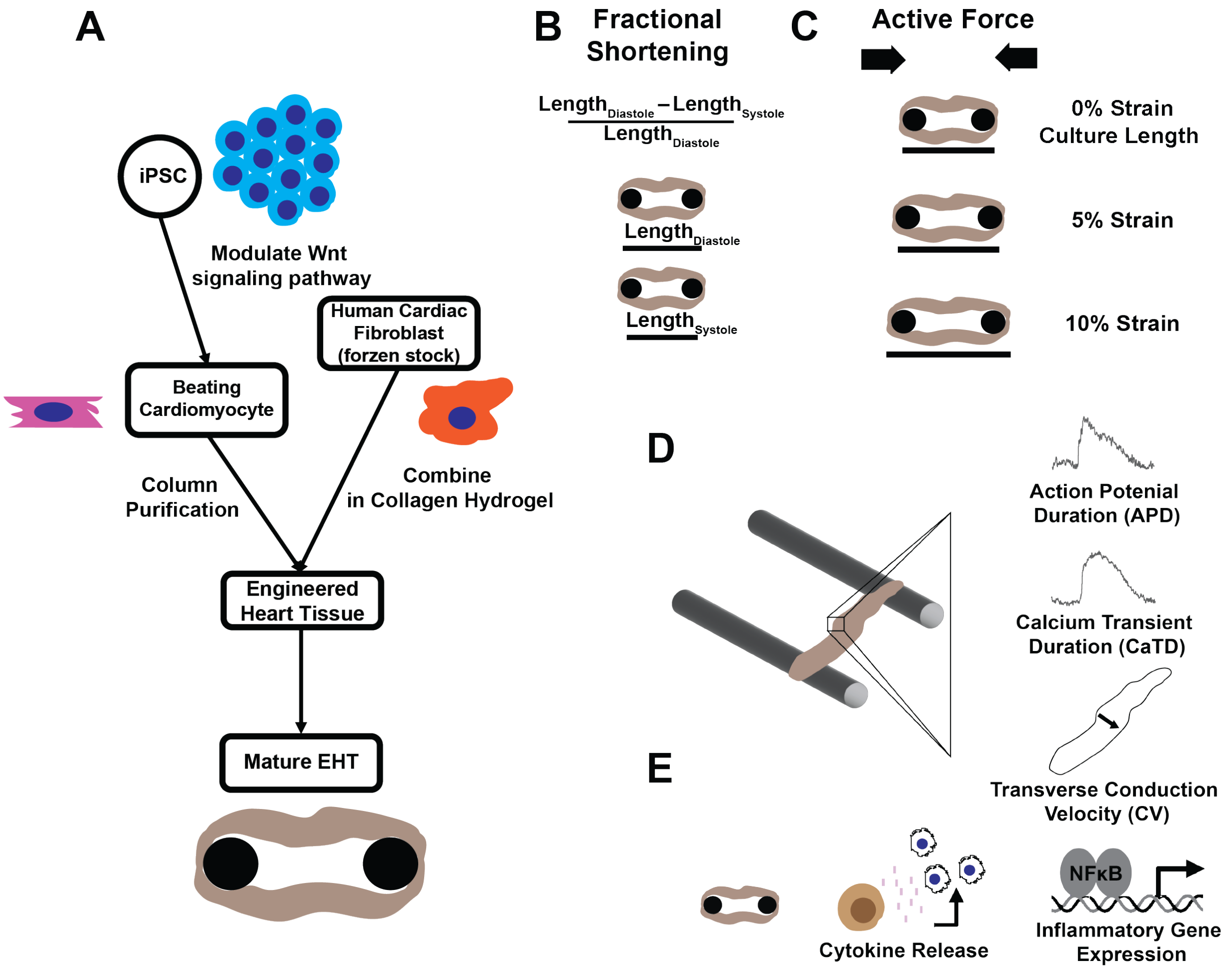

Supplementary Figure 1. Experimental overview. **A**) Human induced pluripotent stem cell-derived cardiomyocytes (hiPSC-CMs) were differentiated. hiPSC-CMs were purified and combined with human cardiac fibroblasts from frozen stock in a 3D collagen hydrogel to form engineered heart tissues (EHTs). **B-E**) EHTs with *DSP* pathogenic variants were subject to electrical, mechanical, and immune phenotyping. **B**) Fractional shortening measurements were calculated based on auxotonic contraction observed using optical microscopy. **C**) Active force measurements made using force transducer under paced conditions. Transducer distance was increased to simulate strain differences compared to baseline. **D**) Optical mapping of EHTs in the transverse plane allowed for simultaneous assessment of Action Potential Duration (APD), Ca^{2+} Transient Duration (CaTD) and Transverse Conduction Velocity (CV) across tissues under paced conditions. **E**) Immune phenotyping was performed used media from EHTs incubated with an immunoblot of secreted cytokines and inflammatory gene expression as measured by qRT-PCR.

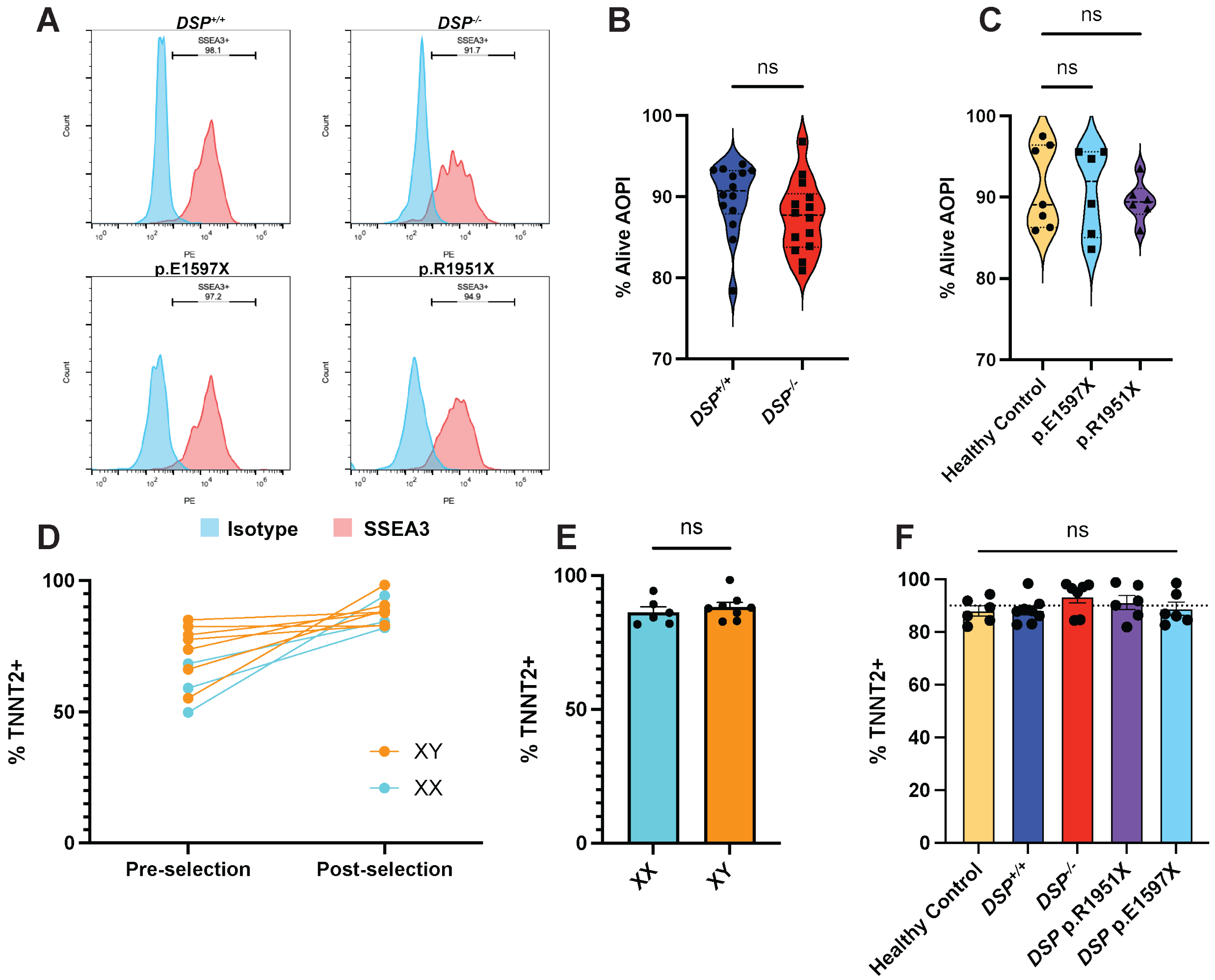

Supplementary Figure 2. Quality control metrics for hiPSC-CM quality during EHT production. A) Representative staining for stage-specific embryonic antigen-3 (SSEA-3) compared to isotype control as measured via flow cytometry for identified hiPSC lines generated for study. Passages were maintained at greater than 90% SSEA-3^{+} to ensure proper differentiation potential. **B**) Cell viability assay using acridine orange and propidium iodide (AOPI) indicated no significant difference in cell death between *DSP*$^{-/-}$ and *DSP*$^{+/+}$ hiPSC-CM monolayers when measured prior to column purification (Welch's t-test, n = 14 per condition). **C**) AOPI cell viability assay indicates no significant difference in cell death between heterozygous p.E1597X and p.R1951X hiPSC-CM monolayers and healthy control when measured prior to column purification (2-way ANOVA, n = 6 per condition). **D**) Representative flow cytometry data measuring cardiac troponin staining (TNNT2) of hiPSC-CM monolayers before and after column purification among control XX and XY hiPSC lines (XX = 3, XY =6). **E**) End-stage troponin staining for column-purified hiPSC-CMs derived from XX and XY iPSC cell lines indicates no significant differences in final purity between cell lines during EHT manufacturing (Welch's t-test, n = 6 per condition). **F**) Quality control measurements taken prior to EHT fabrication indicate no significant difference between hiPSC lines used throughout the study as measured via TNNT2^{+} (n = 6-8 per condition, 2-way ANOVA). Passages with purity less than 90% TNNT2^{+} were not pursued for further experimentation.

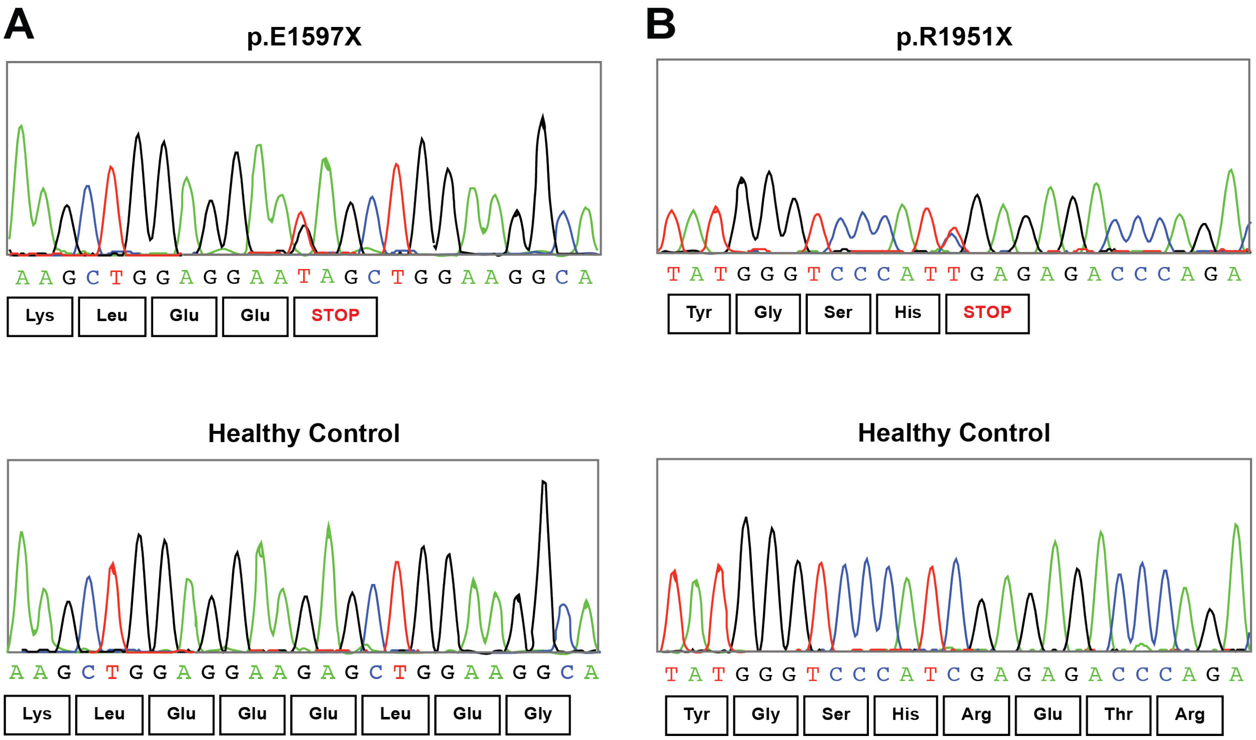

Supplementary Figure 3. Sanger sequencing confirms *DSP* truncating variations in hiPSC lines derived from patients. A) Representative sense sequence for *DSP* p.E1597 locus in exon 23 of *DSP* (hg19, chr6:7,581,200-7,581,224) demonstrating c.4789 G>T mutation resulting in p.E1597X truncating mutation in reading frame. Chromatogram for Healthy Control hiPSC line shown for reference. **B)** Representative sense sequence for *DSP* p.R1951 locus in exon 24 of *DSP* (hg19, chr6:7,583,340-7,583,378) with c.5851 C>T mutation resulting in p.R1951X truncation in reading frame. Chromatogram for Healthy Control hiPSC line shown for reference.

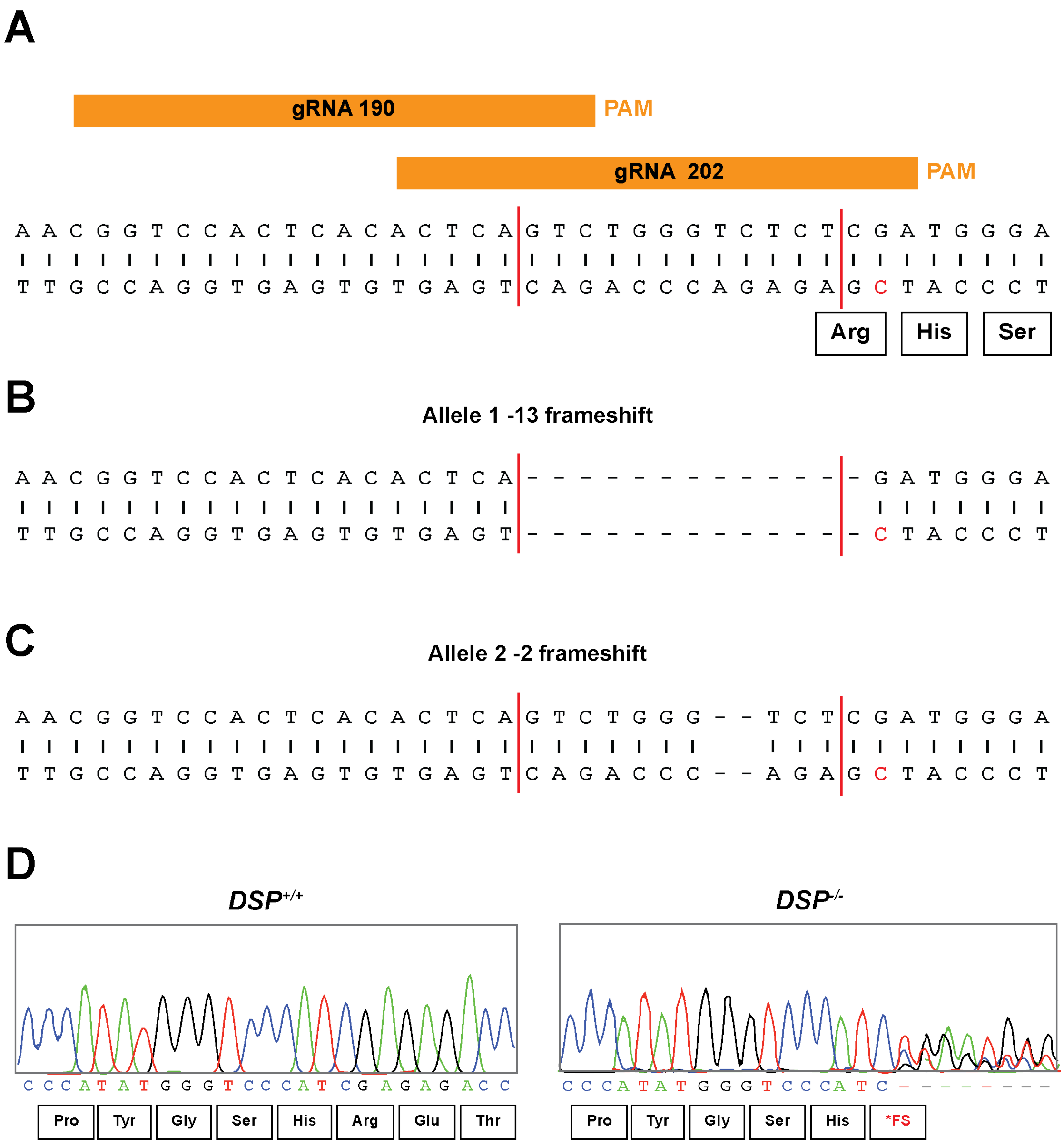

Supplementary Figure 4. CRISPR-Cas9 gRNAs targeting *DSP* to generate *DSP*$^{-/-}$ line. A) Representative antisense sequence for *DSP* exon 24 (hg19, chr6:7,583,340-7,583,378) with gRNA 190 and gRNA 202 (Supplementary Table 1) in alignment with respective protospacer adjacent motifs (PAMs). Guides were designed to target the c.5851, which was the region in the patient derived p.R1951X cell line (wildtype c.5851 C in red). Predicted cut sites of Cas9 nuclease indicated by red lines. **B**) Sequencing results of reference locus with -13 base pair deletion c.5852del13 as confirmed by next generation sequencing (NGS)**. C**) Sequencing results of reference locus with -2 base pair deletion c.5856del2 as confirmed by NGS. **D**) Sanger sequencing chromatograms of *DSP* p.R1951 locus (sense) from resultant biallelic frameshift mutant *DSP*$^{-/-}$ hiPSC line compared to isogenic *DSP*$^{+/+}$ from which it was derived.

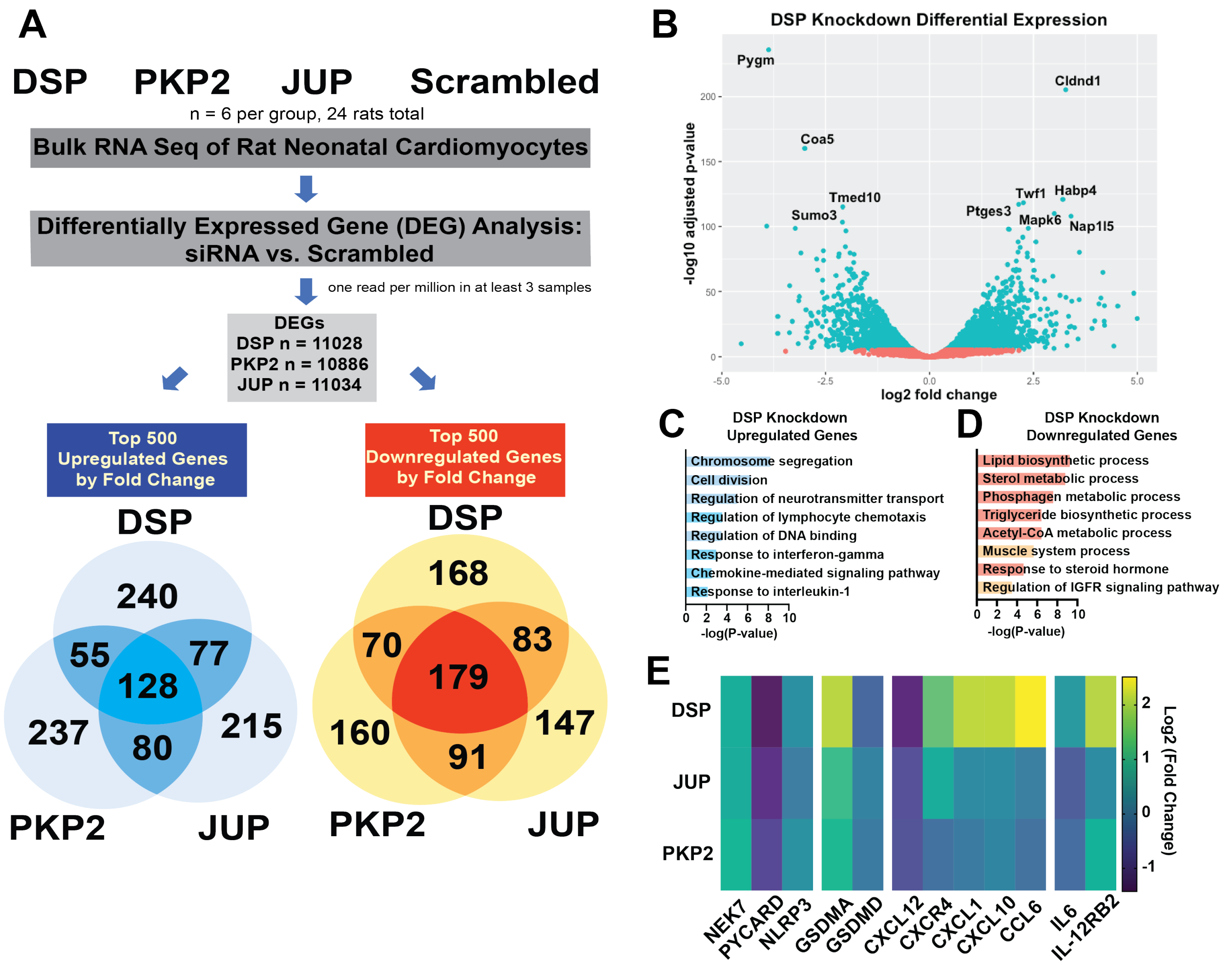

Supplementary Figure 5. Short interfering RNA (siRNA) treatment of neonatal rat ventricular myocytes (NRVMs) demonstrating upregulation of innate immunity with *Dsp* reduction. **A**) Experimental overview of bulk RNA-sequencing analysis for NRVMs treated with siRNA targeting desmoplakin (*Dsp*), plakophilin-2 (*Pkp2*) and plakoglobin (*Jup*). Differentially expressed genes (DEGs) were identified comparing siRNA-treated conditions with scrambled control. Comparative analysis identified 128 genes commonly upregulated and 179 genes commonly downregulated across all three treatment groups (n = 6 rats per condition). **B**) Volcano plot for NRVMs treated with siRNA targeting *Dsp* compared to scrambled control identifying the top 10 DEGs as measured by p-value. **C**). Gene ontology (GO) analysis of top 500 upregulated DEGs in *Dsp* knockdown cells identified pathways associated with immune response and chemokine signaling (light blue). **D**) GO enrichment of top 500 downregulated DEGs in *Dsp* knockdown cells indicated reduction in transcript for pathways involved in lipid metabolism and steroid signaling (light orange). **E**) Heat map of commonly upregulated genes associated with inflammatory GO terms across *Dsp*, *Jup* and *Pkp2* knockdown treatment groups indicated that *Dsp*-deficient NRVMs had significantly higher expression of genes related to chemotaxis and innate immune activation compared to NRVMs deficient in other components of the desmosome.

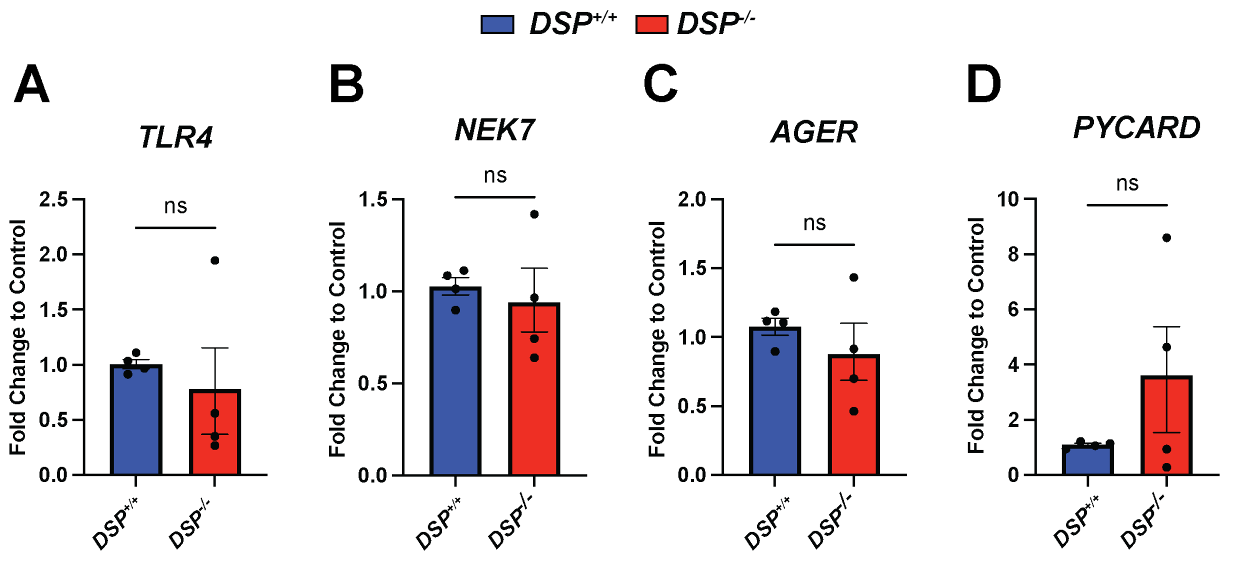

Supplementary Figure 6. *DSP*$^{-/-}$ EHTs express similar levels of innate immune receptors compared to isogenic *DSP*$^{+/+}$ control. **A**) qPCR analysis of Toll-like receptor 4 (*TLR4*) a pattern recognition receptor (PRR) activated by lipopolysaccharide (LPS) and other pathogen associated molecular patterns, showing no significant difference between *DSP*$^{-/-}$ and *DSP*$^{+/+}$ controls. **B**) qPCR of NIMA-related kinase 7 (*NEK7*), a mammalian serine-threonine kinase that forms a component of the intracellular inflammasome, showing no significant difference in expression levels between *DSP*$^{-/-}$ and *DSP*$^{+/+}$ EHTs. **C**) qPCR of receptor for advanced glycation endproducts, encoded by *AGER*, a transmembrane PRR known to bind high mobility group protein B1 (HMGB1), showing no significant difference between *DSP*$^{-/-}$ and *DSP*$^{+/+}$ controls. **D**) qPCR levels of transcript for *PYCARD*, encoding the protein Apoptosis-associated Speck-like protein containing a CARD domain (ASC) that participates in caspase recruitment and IL-1β processing, with no significant difference between *DSP*$^{-/-}$ EHTs and isogenic controls (Welch's t-test, n = 4 per condition).

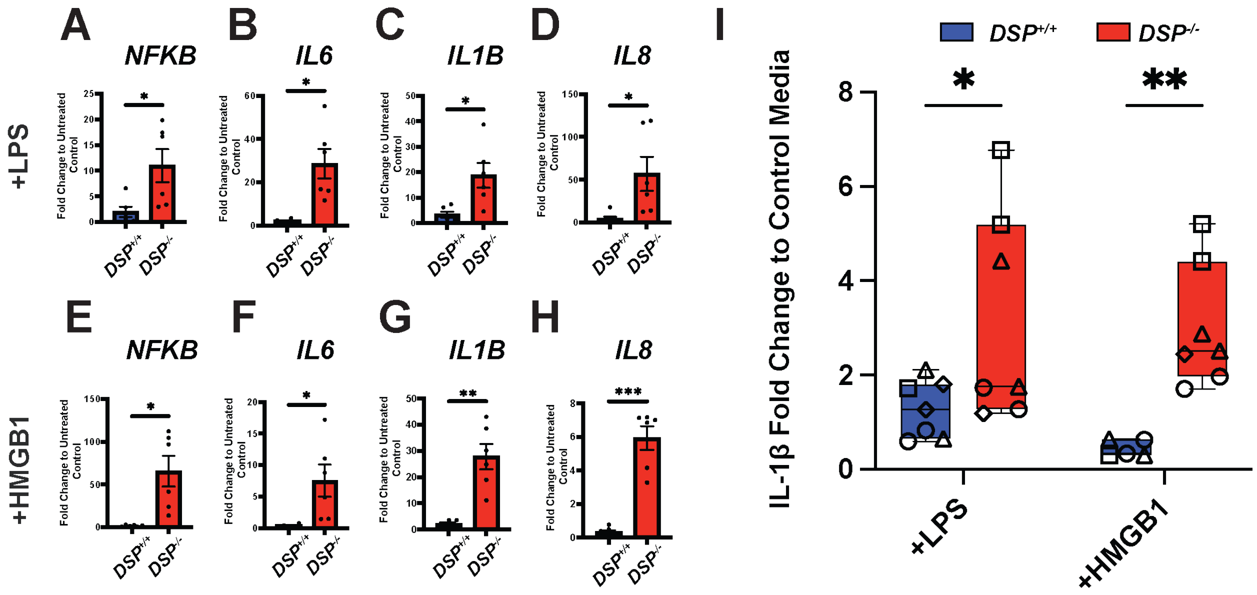

Supplementary Figure 7. Hyperresponsive innate immune activation in *DSP*$^{-/-}$ EHTs. A-H) qRT-PCR of *DSP*$^{-/-}$ EHTs compared to isogenic control demonstrated significant elevation of transcripts of genes implicated in innate immune activation including *NFKB* (**A**, **E**), *IL6* (**B**, **F**), *IL1B* (**C**, **G**) and *IL8* (**D**, **H**) following 48 hours of LPS (**A-D**) or HMGB1 (**E-H**) exposure compared transcript level in control media (*< 0.05, **<0.01, ***<0.001, for 2-tailed t-test with Welch's correction; *n* = 6 per genotype; data reflects three independent differentiations). **I**) Shown is fold induction of IL-1β released into EHT media after LPS and HMGB1 exposure (48h) compared to baseline control media. *DSP*$^{-/-}$ EHTs have greater cytokine elevation compared to *DSP*$^{+/+}$ EHTs (*< 0.01, **<0.001 for 2-way ANOVA, n = 7 per genotype; data reflects four independent batches).

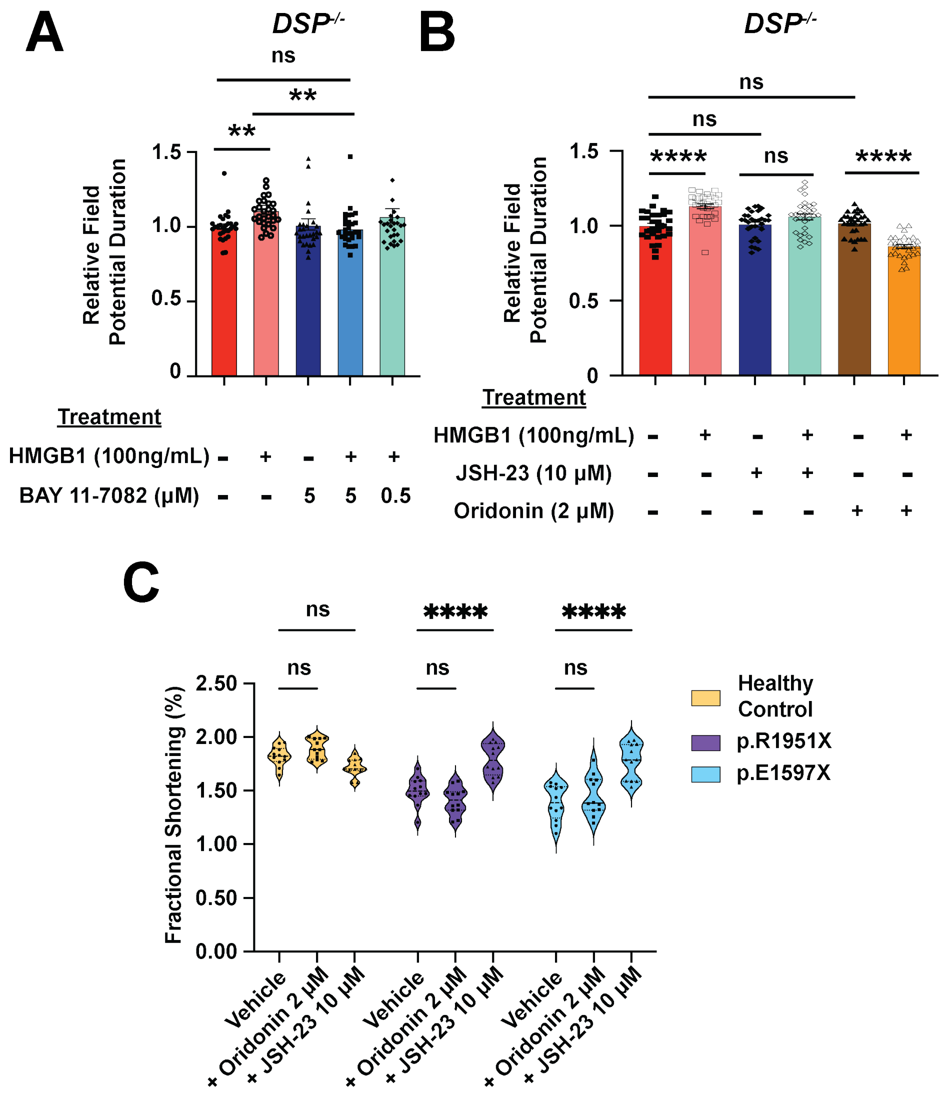

Supplementary Figure 8. NFκB inhibition attenuates inflammatory impacts on cardiac function in *DSP*tv models. A) Multielectrode array (MEA) measurements of $DSP^{-/-}$ monolayers treated with 100 ng/mL of HMGB1 demonstrate a significant increase in FPD compared to untreated control media that is not observed with the addition of NFκB inhibitor BAY 11-7082 (**< 0.01 for 2-tailed t-test with Welch's correction, n = 29 per condition, data reflects one independent differentiation and replicated across three independent differentiations). **B**) MEA measurements of $DSP^{-/-}$ hiPSC-CMs grown as monolayers treated with 100 ng/mL of HMGB1 demonstrate a significant increase in FPD compared to vehicle treatment. With the addition of the NFκB inhibitor JSH-23, this prolongation of FPD did not occur. The NLRP3 inflammasome inhibitor oridonin (Ori) shortened the FPD induced by HMGB1 (****< 0.001 for 2-tailed t-test with Welch's correction, n = 30 per condition, data reflects one independent differentiation and replicated across three independent differentiations). **C**) Fractional shortening measurements of patient-derived heterozygous *DSP*tv p.E1597X and p.R1951X EHTs treated with JSH-23 and Ori demonstrated that NFκB inhibition improved contractility at baseline culture conditions. This improvement in contractility was not observed in Ori-treated EHTs or in Healthy Control EHTs (****< 0.001 for 2-way ANOVA, n = 4 per condition, data reflects one independent differentiation). All data presented as mean ± SEM.

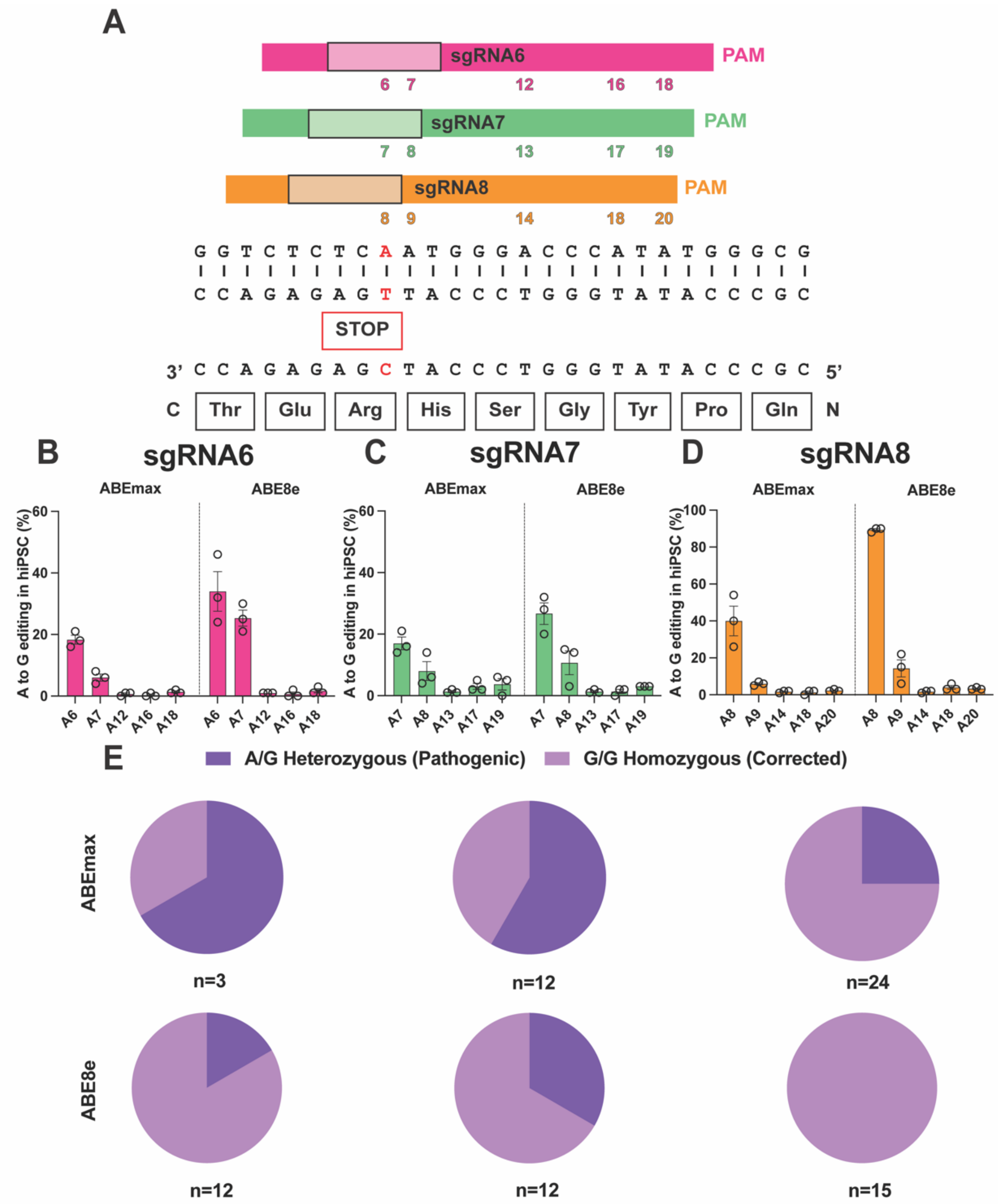

Supplementary Figure 9. Correction of *DSP* p.R1951X using adenine base editing (ABE) **A)** Representative antisense sequence for *DSP* p.R1951X locus in exon 24 of DSP (hg19, chr6:7,583,340-7,583,378) with predicted sgRNAs (**Supplementary Table 7**) targeting the pathogenic variant c.5851 C>T (antisense c.5851 G>A) using three potential protospacer adjacent motifs (PAMs). Guides were designed with optimal base editing window in darkened box (positions 4-8). Pathogenic variant c.5851 G>A in red. Reading frame indicates predicted truncation point in full length amino acid sequence. **B-D**) Percentage of adenine (A) to guanine (G) editing in human iPSCs (hiPSC) for each adenine in sgRNA6 (**B**), sgRNA7 (**C**), and sgRNA8 (**D**) after base editing with ABEmax or ABE8e in p.R1951X hiPSCs, as determined by Sanger sequencing (n = 3 per condition). **E**) Sanger sequencing results of individual clones treated as in **B-D** with resultant genotype indicating outcomes of A-to-G conversion of c.5851 to Corrected Allele or Pathogenic Allele of p.R1951X hiPSCs (ABEmax: sgRNA6 = 3, sgRNA7 = 12, sgRNA8 = 24; ABE8e: sgRNA6 = 12, sgRNA7 = 12, sgRNA8 = 15).

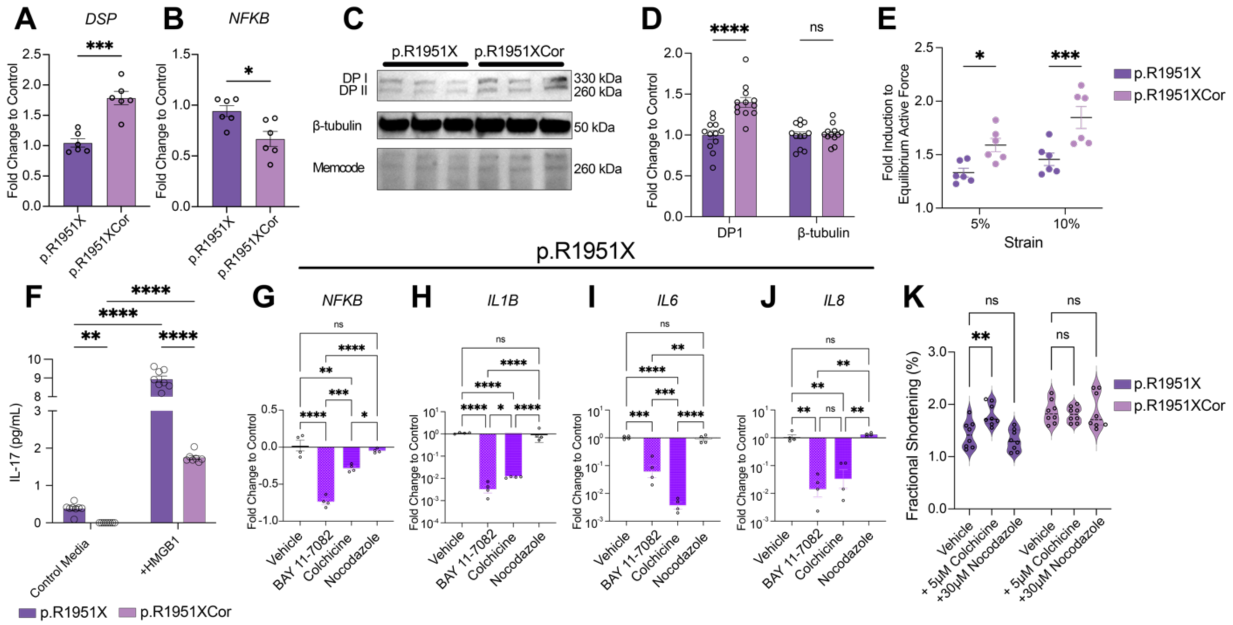

Supplementary Figure 10. Restoration of desmoplakin content in p.R1951XCor EHTs attenuates mechanical benefits of anti-inflammatory therapy. A-B) RT-qPCR of *DSP* p.R1951XCor EHTs confirms that genomic correction results in significant upregulation of *DSP* transcript (**A**) with concomitant reduction in *NFKB* mRNA (**B**) compared to *DSP* p.R1951X when cultured at baseline conditions. (*< 0.05, ***<0.001, for 2-tailed t-test with Welch's correction; *n* = 6 per condition, data reflects three independent batches). **C-D**) Immunoblot of *DSP* p.R1951XCor EHTs demonstrated significant increase in DPI compared to isogenic control EHTs (p.R1951X), (****< 0.0001 for 2-way ANOVA; *n* = 12 per condition, data reflects three independent batches). **E**) Genomic correction improve active force production with 5% and 10% strain, indicating enhanced contractile reserve (*< 0.05, ***<0.001 for 2-way ANOVA; *n* = 6 EHTs per condition, data reflects three independent batches. Data presented as average active force value normalized to average baseline measurement per EHT). **F**) IL-17 release into EHT media was reduced in p.R1951Cor vs p.R1951X at baseline and in response to 48hrs of HMBG1 treatment (**< 0.01, ****<0.0001 for 2-way ANOVA, n = 8 EHTs per condition). **G-J**) RT-qPCR of *DSP* p.R1951X EHTs following 48hrs exposure to NFκB inhibitor BAY 11-7082, colchicine, or microtubule inhibitor nocodazole, indicated significant reduction in transcripts for *NFKB* (**G**), *IL1B* (**H**), *IL6* (**I**) and *IL8* (**J**) in response to colchicine and BAY 11-7082 but not nocodazole (*<0.05, **< 0.01, ***<0.001, ****<0.0001 for 2-way ANOVA, n = 4 EHTs per condition). **K**) Fractional shortening of p.R1951X EHTs treated with 5 μM colchicine for 48 hrs demonstrated improved contractility compared to vehicle control with no significant change in response to 30 μM nocodazole over the same time course. Changes in contractility were not observed in p.R1951XCor EHTs treated similarly (**< 0.001, for 2-way ANOVA, n = 8 EHTs per condition). All data presented as mean ± SEM.
