# Supplementary material for: Susceptibility to innate immune activation in genetically mediated myocarditis
Source: J Clin Invest. 2024 May 16;134(13):e180254. doi: 10.1172/JCI180254 (PMC11213508; doi:10.1172/JCI180254)

Figure 1D

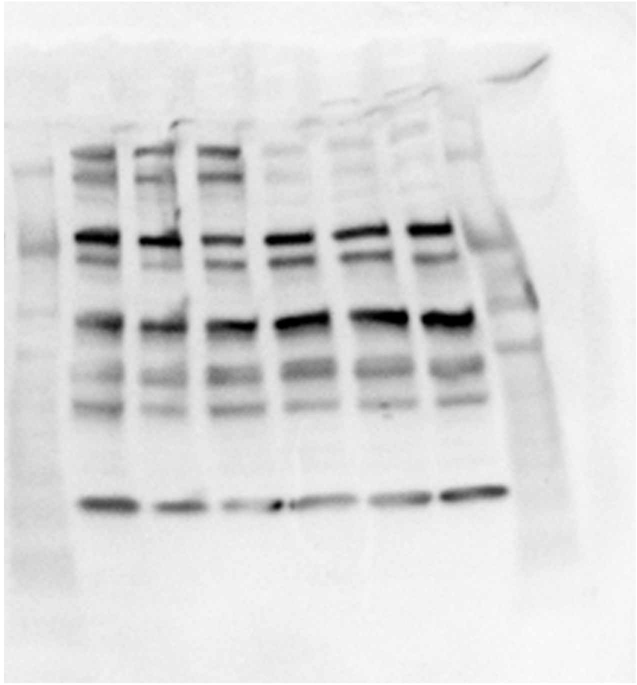

Figure 1D

Cxn43 43 kDa

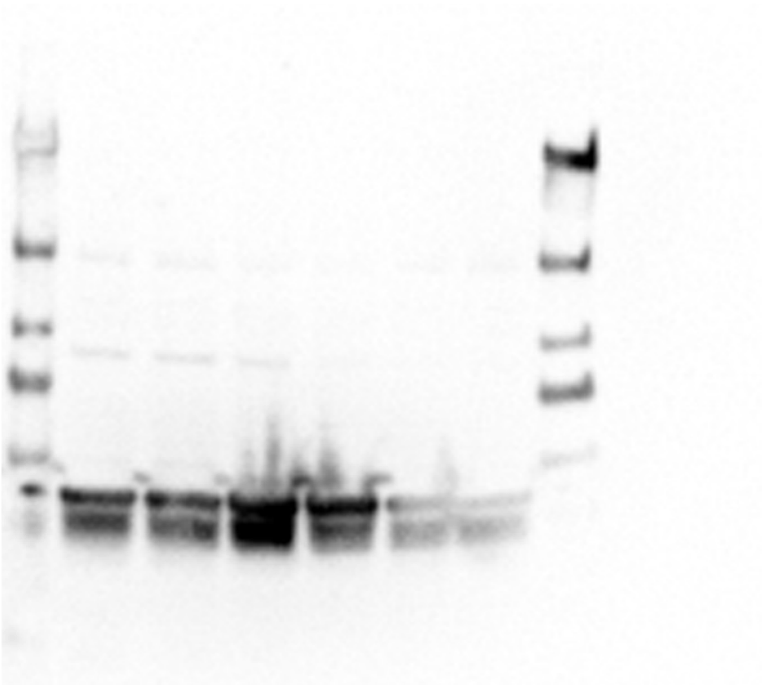

Figure 1D

Memcode 260 kDa

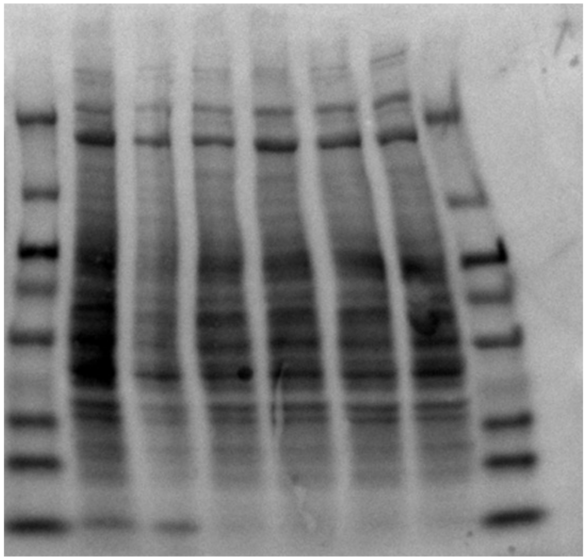

Figure 3I

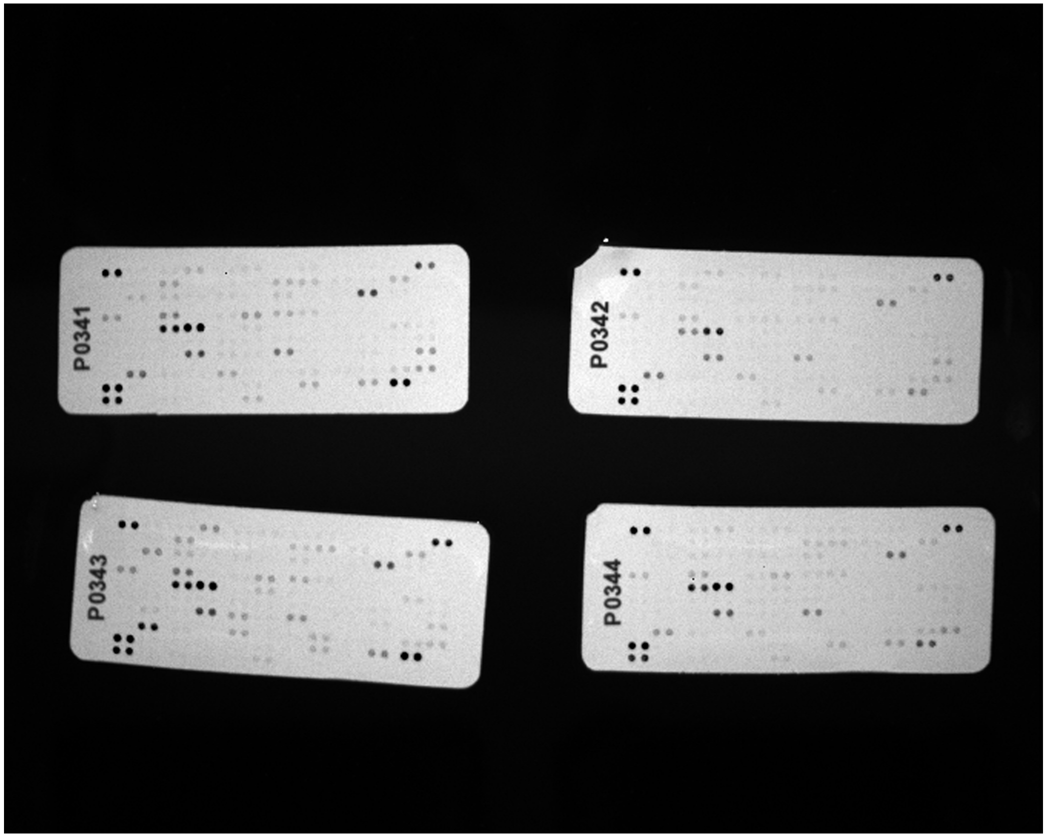

Figure 6D

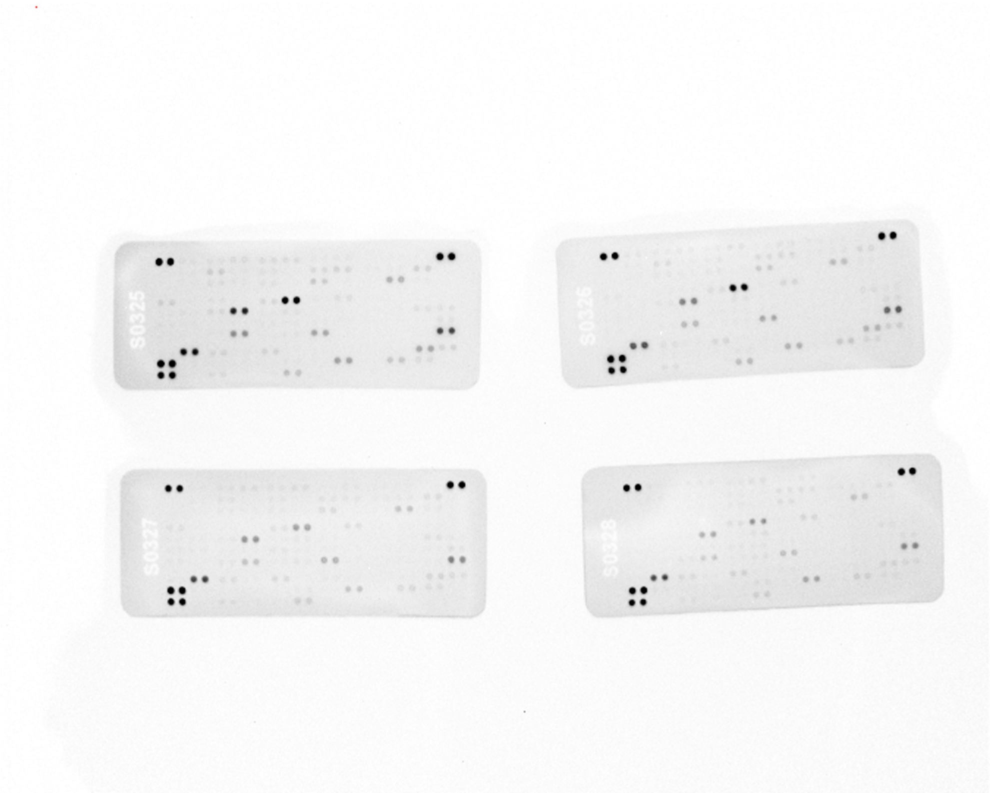

Figure 7G

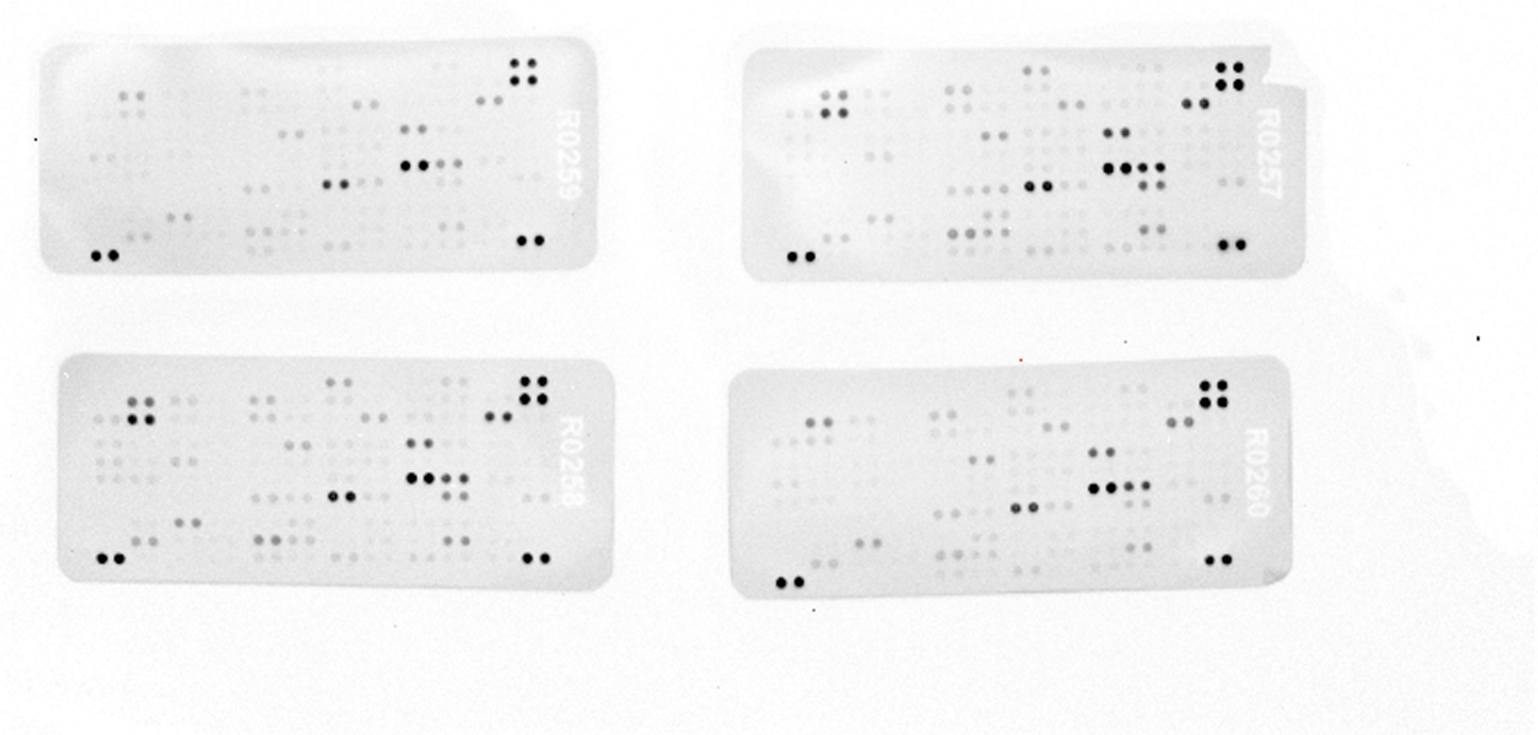

Figure 8C

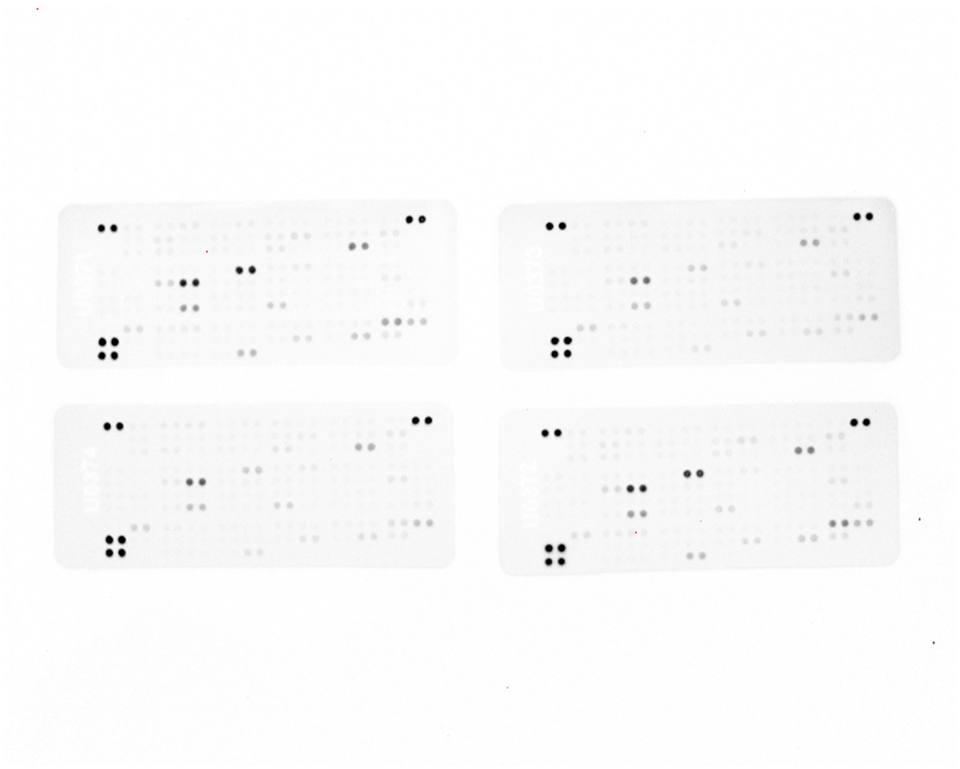

Supplemental Figure 10B

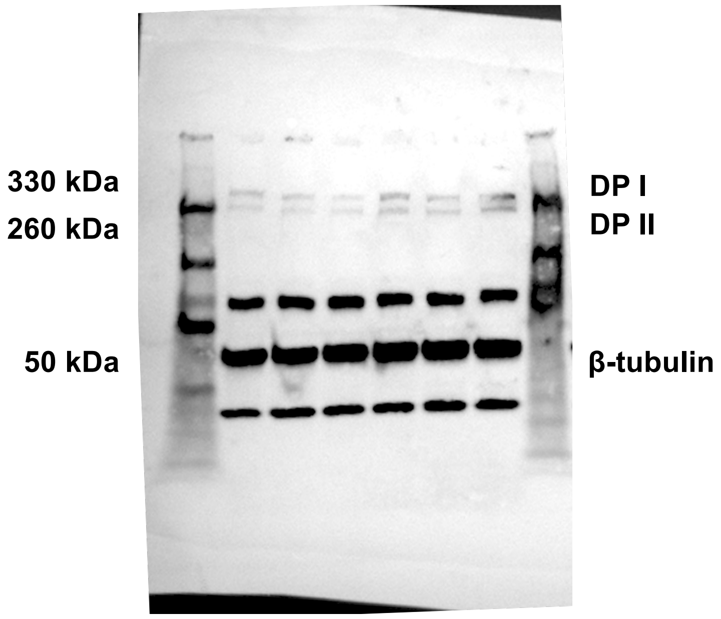

Supplemental Figure 10B

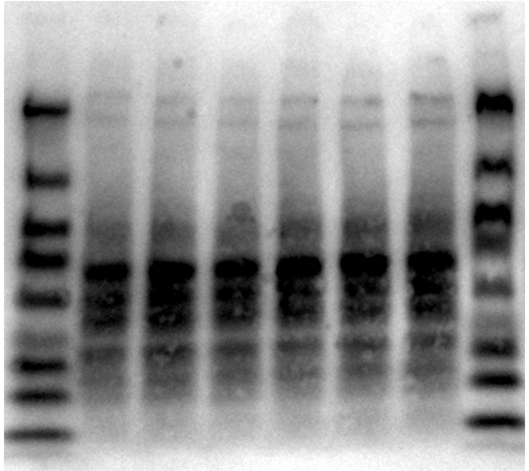

Supplement: Unedited blot and gel images [file jci-134-180254-s252.pdf]
